# USP15 negatively regulates lung cancer progression through the TRAF6-BECN1 signaling axis for autophagy induction

**DOI:** 10.1038/s41419-022-04808-7

**Published:** 2022-04-14

**Authors:** Mi-Jeong Kim, Yoon Min, Soo-Kyung Jeong, Juhee Son, Ji Young Kim, Ji Su Lee, Duk-Hwan Kim, Joo Sang Lee, Eunyoung Chun, Ki-Young Lee

**Affiliations:** 1 Department of Immunology, Sungkyunkwan University School of Medicine, Suwon, 16419 Republic of Korea; 2 R&D Center, CHA Vaccine Institute, Seongnam-si, 13493 Republic of Korea; 3 Department of Molecular Cell Biology, Sungkyunkwan University School of Medicine, Suwon, 16419 Republic of Korea; 4 Samsung Biomedical Research Institute, Sungkyunkwan University School of Medicine, Suwon, 16419 Republic of Korea; 5 Department of Precision Medicine, Sungkyunkwan University School of Medicine, Suwon, 16419 Republic of Korea; 6 Department of Health Sciences and Technology, Samsung Advanced Institute for Health Sciences & Technology, Samsung Medical Center, Sungkyunkwan University, Seoul, 06351 Republic of Korea; 7 Single Cell Network Research Center, Sungkyunkwan University School of Medicine, Suwon, 16419 Republic of Korea

**Keywords:** Non-small-cell lung cancer, Cell invasion

## Abstract

TNF receptor-associated factor 6 (TRAF6)-BECN1 signaling axis plays a pivotal role in autophagy induction through ubiquitination of BECN1, thereby inducing lung cancer migration and invasion in response to toll-like receptor 4 (TLR4) stimulation. Herein, we provide novel molecular and cellular mechanisms involved in the negative effect of ubiquitin-specific peptidase 15 (USP15) on lung cancer progression. Clinical data of the TCGA and primary non-small cell lung cancer (NSCLC) patients (*n* = 41) revealed that the expression of USP15 was significantly downregulated in lung cancer patients. Importantly, *USP15*-knockout (*USP15*KO) A549 and *USP15*KO H1299 lung cancer cells generated with CRISPR-Cas9 gene-editing technology showed increases in cancer migration and invasion with enhanced autophagy induction in response to TLR4 stimulation. In addition, biochemical studies revealed that USP15 interacted with BECN1, but not with TRAF6, and induced deubiquitination of BECN1, thereby attenuating autophagy induction. Notably, in primary NSCLC patients (*n* = 4) with low expression of *USP15*, 10 genes (*CCNE1, MMP9, SFN, UBE2C, CCR2, FAM83A, ETV4, MYO7A, MMP11,* and *GSDMB*) known to promote lung cancer progression were significantly upregulated, whereas 10 tumor suppressor genes (*FMO2, ZBTB16, FCN3, TCF21, SFTPA1B, HPGD, SOSTDC1, TMEM100, GDF10*, and *WIF1*) were downregulated, providing clinical relevance of the functional role of USP15 in lung cancer progression. Taken together, our data demonstrate that USP15 can negatively regulate the TRAF6-BECN1 signaling axis for autophagy induction. Thus, USP15 is implicated in lung cancer progression.

## Introduction

Autophagy is a functional response to a variety of cellular conditions, such as genomic damage, metabolic stress, infections, and tumorigenesis [1–6]. It has been reported that toll-like receptor (TLR)-mediated signaling plays a pivotal role in cancer progression through autophagy induction and the production of cytokines such as interleukin-6 (IL-6), chemokine (C-C motif) ligand 2 (CCL2/MCP-1), chemokine (C-C motif) ligand 20 (CCL20/MIP-3α), vascular endothelial growth factor A (VEGFA), and matrix metallopeptidase 2 (MMP2) [7]. In terms of molecular mechanisms, these cellular events are critically dependent on TNF receptor-associated factor 6 (TRAF6) signaling [7]. TRAF6, an E3 ubiquitin-protein ligase and adaptor protein, can functionally regulate downstream signaling cascades for the production of cytokines through the activation of the nuclear factor of kappa light polypeptide gene enhancer in B-cells (NF-κB) and the induction of autophagy through ubiquitination of beclin 1 (BECN1) [7–12]. Recent studies have demonstrated that interruption of TRAF6-BECN1 signaling induced by TLR4 can lead to inhibition of autophagy induction, resulting in attenuated cancer migration and invasion [10–12]. This suggests that the TRAF6-BECN1 signaling axis plays a pivotal role in cancer progression through autophagy induction.

Deubiquitinating enzymes (DUBs) can counteract cellular E3 ubiquitin ligases by removing ubiquitin from substrates, thereby regulating a variety of cellular signaling processes [13]. Ubiquitin-specific peptidase 15 (USP15) is a member of the largest subfamily of cysteine protease DUBs [14, 15]. We have previously reported that charged multivesicular body protein 5 (CHMP5) can cooperate with a PDB genetic risk factor valosin-containing protein (VCP/p97) to stabilize the inhibitor of NF-κB, thus downregulating ubiquitination of IκBα via the deubiquitinating enzyme USP15 [16]. Furthermore, USP15 can antagonize parkin-mediated mitochondrial ubiquitination and mitophagy [17]. Knockdown of USP15 can rescue the mitophagy defect in fibroblasts of patients with Parkinson’s disease (PD) and increase their parkin levels [17]. Although much research progress has been made in exploring the roles of DUBs in various human diseases [13], we know little about the molecular and cellular mechanisms by which USPs are implicated in cancer progression. Thus, this study explores the molecular and cellular mechanism by which USP15 is implicated in cancer progression through the regulation of the TRAF6-BECN1 signaling axis for autophagy induction.

TCGA data analysis revealed that five different cancer types (LUAD, LUSC, OV, SKCM, and TGCT) showed significant downregulation of USP15, whereas no significant difference in expression of USP15 was seen in the other 28 different cancer types. Notably, the downregulation of USP15 was significant in primary non-small cell lung cancer (NSCLC) patients, especially in patients with lung adenocarcinoma. Based on these findings and previous reports [16, 17], we investigated whether USP15 as a deubiquitinating enzyme was implicated in lung cancer progression, especially through the regulation of TRAF6-BECN1 signaling axis for autophagy induction. We found that USP15 interacted with BECN1, but not with TRAF6, and induced deubiquitination of BECN1. Importantly, *USP15*-knockout (*USP15*KO) A549 and *USP15*KO H1299 lung cancer cells generated by CRISPR-Cas9 gene editing showed increases of cancer migration and invasion accompanied by enhanced autophagy induction in response to TLR4 stimulation. These results strongly support that USP15 is negatively implicated in lung cancer progression through the regulation of autophagy induction. Regarding clinical aspects, primary LUAD patients (*n* = 4) with low expression of USP15 showed significant upregulation of genes (*n* = 10) involved in the promotion of lung cancer proliferation, migration, invasion, and metastasis, whereas tumor suppressor genes (*n* = 10) were downregulated. These results strongly suggest that USP15 is negatively implicated in lung cancer progression through the TRAF6-BECN1 signaling axis by regulating autophagy induction.

## Materials and methods

### Patient-derived non-small cell lung cancer samples (NSCLC)

Tumor and matched normal tissues were obtained from 41 patients with primary NSCLC in accordance with the ethical principles stated in the Declaration of Helsinki. This study was approved by the Institutional Review Board (IRB) of Samsung Medical Center (SMC) (IRB#: 2010-07-204), following procedures previously described [18, 19]. We obtained written informed consent from each patient before surgery for using their pathological specimens for research use.

### Cells

Human embryonic kidney (HEK) 293T cells (ATCC, CRL-11268) were cultured and maintained in Dulbecco’s modified Eagle’s medium (DMEM; Thermo Fisher Scientific, 11965092) with 10% fetal bovine serum (FBS). A549 cells (human lung cancer cell line; ATCC, CCL-185) and H1299 cells (human non-small cell lung cancer cell line; ATCC, CRL-5803) were maintained in RPMI 1640 medium (Sigma-Aldrich, 31800-022) supplemented with 10% FBS, penicillin (100 μg/mL), and streptomycin (100 μg/mL) in a 5% CO_2_ humidified atmosphere at 37 °C.

### Antibodies and reagents

Anti-MYC (2276), anti-GAPDH (2118), and anti-LC3A/B (4108) were purchased from Cell Signaling Technology. Anti-FLAG (SAB4200071) was purchased from Sigma-Aldrich. Anti-HA (ab18181) and anti-USP15 (ab97533) were purchased from Abcam. Lipopolysaccharide (LPS; serotype 0128: B12), chloroquine (CQ; C6628), dimethyl sulfoxide (DMSO; 472301), puromycin (P8833), paraformaldehyde (P6148), Triton X-100 (T8787), 3-methyladenine (3-MA; M9281), gentamicin (G1272), deoxycholate (D6750), and Dulbecco’s phosphate-buffered saline (DPBS; D8537) were purchased from Sigma-Aldrich. Lipofectamine 2000 (11668019) was purchased from Thermo Fisher Scientific.

### Plasmid constructs

FLAG-TRAF6 (21624), FLAG-HA-USP15 (22570), and FLAG-BECN1 (24388) plasmids were purchased from Addgene. HA-tagged Ub plasmids were obtained from Dr. J. H. Shim (University of Massachusetts Medical School, USA). Using the FLAG-HA-USP15 plasmid, full-length FLAG-USP15 and MYC-USP15 constructs were cloned into a pCMV-3Tag-7 (Agilent technologies, 240202) or a pCMV-3Tag 6 vector (Agilent technologies, 240200). Using FLAG-BECN1, the full-length MYC-BECN1 construct was cloned into a pCMV-3Tag-7 vector (Agilent Technologies, 240202). Truncated mutants of MYC-BECN1 were generated as previously described [10, 20–23]. MYC-USP15 C269A and MYC-USP15 H862A mutants were generated by site-directed mutagenesis as previously described [24].

### Generation of USP15-knockout cancer cell lines by CRISPR/Cas9

Guide RNA sequences for CRISPR/Cas9 were designed as 5’-CACCG CAGTTGGGACAAATACCAGATGG-3’ and 3’-CGTCAACCCTGTTTATGGTCTACC CAAA-5’ for human USP15. *USP15*KO A549 and *USP15*KO H1299 cancer cells were generated as previously described [11, 12, 25]. Complementary oligonucleotides to guide RNAs (gRNAs) were annealed and cloned into a lentivirus CRISPR v2 vector (Addgene plasmid, 52961). Lenti CRISPR v2/gRNA was transfected into A549 or H1299 cells using Lipofectamine 2000 according to the manufacturer’s instructions. *USP15*KO A549 and *USP15*KO H1299 cell colonies were selected as previously described [11, 12, 25] and confirmed by western blots.

### Western blotting (WB) and immunoprecipitation (IP) assays

WB and IP assays were performed as previously described [10, 20–23]. Briefly, HEK 293T cells were seeded into six-well plates, transfected, and treated as described in the text and Figures. These cells were then incubated for 38–48 h. After collecting cells, cell lysates were prepared and immunoprecipitated with anti-MYC and anti-FLAG antibodies. IP complexes were separated by sodium dodecyl sulfate-polyacrylamide gel electrophoresis (SDS-PAGE, 6–10%) and immune-probed with different antibodies as indicated in the text. For ubiquitination assay, mock vector, FLAG-tagged BECN1, MYC-tagged USP15 wild-type (WT), MYC-tagged USP15 C269A mutant, and MYC-tagged USP15 H862A mutant were transfected separately into HEK 293T cells along with HA-tagged Ub as described in the text and Figures. Cell lysates were immunoprecipitated with anti-MYC, anti-HA, or anti-FLAG antibodies and probed with different antibodies as indicated in the text. Control (Ctrl) A549 and *USP15*KO A549 cells were treated with or without the vehicle or CQ (10 μM) or 3-MA (5 mM) in the presence or absence of LPS (10 μg/mL) for 6 h. Cell lysates were immunoblotted with an anti-LC3A/B antibody and anti-GAPDH (as a loading control). For a semi-endogenous IP assay, A549 and H1299 lung cancer cells were transiently transfected with FLAG-USP15. At 48 h post-transfection, cells were treated with or without LPS (10 μg/mL) for 60 min. After collecting cells, cell lysates were prepared and immunoprecipitated with anti-Ig or anti-FLAG antibodies. IP complexes were separated by sodium dodecyl sulfate-polyacrylamide gel electrophoresis (SDS-PAGE, 6–10%) and immune-probed with anti-FLAG and anti-BECN1 antibodies.

### Reverse transcription-quantitative polymerase chain reaction (RT-qPCR) analysis

Control (Ctrl) A549, *USP15*KO A549, Ctrl H1299, and *USP15*KO H1299 cells were treated with or without 10 μg/mL LPS for 6 h. RT-qPCR analysis was performed as previously described [11]. Briefly, after extracting total RNA using an RNA isolation kit (A&A Biotechnology, Gdynia, Poland) according to the manufacturer’s protocol, cDNA was prepared by RT using AmfiRivert II cDNA Synthesis Master Mix (genDEPOT, R550) according to the manufacturer’s protocol. Primers hIL-6 (PPH 00560C), hMMP2 (PPH 00151B), hCCL20 (PPH 00564C-200), and hCCL2 (PPH 00192F) were purchased from Qiagen, Inc. (Chatsworth, CA, USA). Fluorescence detection was performed using an ABI PRISM 7700 Sequence Detector (PerkinElmer; Applied Biosystems; Thermo Fisher Scientific, Inc.). The mRNA expression level was calculated and normalized to the level of *GAPDH*.

### Wound-healing migration assay

A wound-healing migration assay was performed following previous protocols [10–12]. Briefly, Control (Ctrl) A549, *USP15*KO A549, Ctrl H1299, and *USP15*KO H1299 cells were seeded into 12-well plates and cultured to reach confluence. Cell monolayers were gently scratched and washed with culture medium. After floating cells and debris were removed, cells attached to culture plates were treated with vehicle (DMSO), 3-MA (5 mM), or CQ (10 μM) in the presence or absence of LPS (10 μg/mL). Cell images were captured after culturing for different periods as indicated in each experiment.

### Transwell invasion assay

Transwell invasion assay was performed following previous protocols [10–12]. Briefly, Control (Ctrl) A549, *USP15*KO A549, Ctrl H1299, and *USP15*KO H1299 cells were suspended in culture medium (200 μL) without FBS. Cells were then added to the upper compartment of a 24-well Transwell^®^ chamber containing a polycarbonate filter with 8-mm pores and coated with 60 mL of Matrigel (Sigma-Aldrich, E1270; 1:9 dilution). Culture medium with 10% FBS was added to the lower chamber. After incubating for 24 h, cells in the upper compartment were removed, washed with PBS, and fixed. Invaded cells were stained with 4,6-diamidino-2-phenylindole (Sigma-Aldrich, D9542) and quantified by counting the number of fluorescent cells.

### Microarray analysis

Microarray analysis was performed as previously described [18, 19]. From tumor and matched normal tissues of 41 patients with NSCLC, total RNAs were extracted with Trizol (Thermo Fisher Scientific, 15596026) and purified using RNeasy columns (Qiagen, 74106) according to each manufacturer’s protocol. Microarray analysis was performed and analyzed as previously described [18, 25–27].

### TCGA data analysis

The expression of USP15 in human lung cancers (lung adenocarcinoma, LUAD) and (lung squamous cell carcinoma, LUSC) was analyzed using TCGA data (GAPIA, gene expression profiling interactive analysis; http://gepia.cancer-pku.cn/ and http://gepia.cancer-pku.cn/detail.php?gene=USP15).

### Statistical analysis

All in vitro data are expressed as mean ± SEM of triplicate samples. Statistical significance was analyzed with ANOVA or Student’s t-test using GraphPad Prism 5.0 (GraphPad Software, San Diego, CA, USA).

## Results

### USP15 is significantly downregulated in patients with lung adenocarcinoma and negatively implicated in lung cancer invasion

**Fig. 1:**
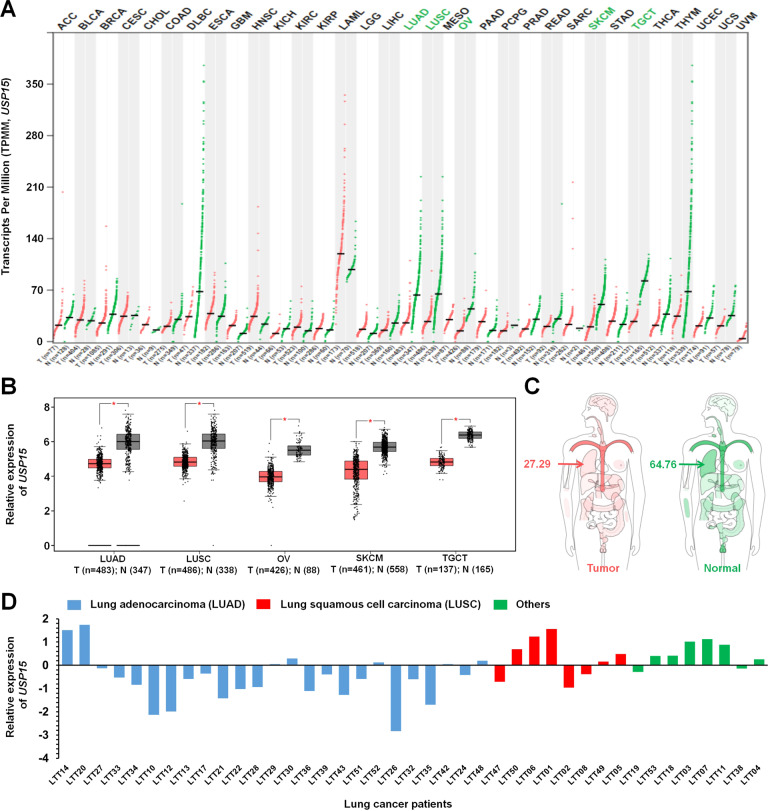
Expression of USP15 is significantly downregulated in lung cancer. **A** The expression of USP15 was analyzed in 33 different cancer types provided by GEPIA (http://gepia.cancer-pku.cn/detail.php?gene=USP15). The expression of USP15 was downregulated in five tumor types (LUAD, LUSC, OV, SKCM, and TGCT) indicated as green. **B** mRNA expression levels of USP15 are significantly downregulated in LUAD tumor (T), LUSC tumor (T), OV tumor (T), SKCM tumor (T), and TGCT tumor (T) tissues than in normal (N) tissues. *P < 0.01 was considered statistically significant. **C** Interactive Bodymap provided by GEPIA shows significant downregulation of USP15 in lung cancer. **D** Microarray analysis was performed for 41 patients with primary NSCLC and matched normal tissues as described in “Materials and methods”. The relative expression of UPS15 is shown. Blue bars, lung adenocarcinoma (LUAD) patients; Red bars, lung squamous cell carcinoma (LUSC) patients; Green bars, other lung cancer patients.

**Fig. 2:**
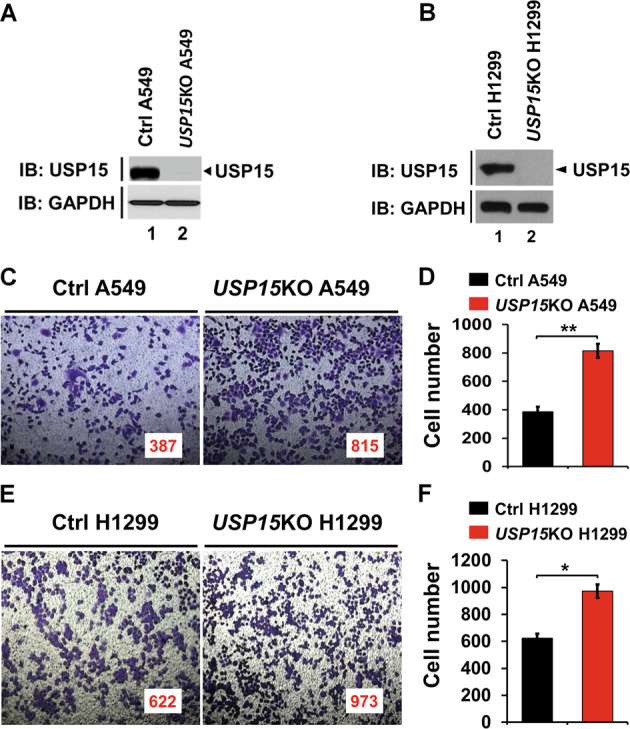
USP15-knockout (*USP15*KO) A549 and *USP15*KO H1299 lung cancers show increases in cancer invasion. **A**, **B** USP15KO A549 (**A**) and USP15KO H1299 (**B**) lung cancer cells were generated using CRISPR/Cas9 gene-editing method as described in “Materials and methods”. *USP15*KO A549 or *USP15*KO H1299 colonies were selected and confirmed by western blots with anti-USP15 or anti-GAPDH antibodies. **C**, **D** Control (Ctrl) A549 and *USP15*KO A549 cells were suspended in culture medium. Cells were added to the upper compartment of a 24-well Transwell® chamber containing polycarbonate filters with 8-mm pores and coated with 60 mL of Matrigel. Culture medium with 10% FBS was added to the lower chamber. After incubating for 24 h. Cells in the upper compartment were removed, washed with PBS, and fixed. Invaded cells were stained with 4,6-diamidino-2-phenylindole (**C**) and quantified by counting the number of cells (**D**). Results are presented as mean ± SEM of three independent experiments. **P < 0.01. **E**, **F** Ctrl H1299 and *USP15*KO H1299 cells were suspended in culture medium. Cells were added to the upper compartment of a 24-well Transwell® chamber. An invasion assay was then performed. Cells were fixed and stained (**E**). The number of migrated cells was counted (**F**). Results are presented as mean ± SEM of three independent experiments. *P < 0.05.

To address the functional role of USP15 in human cancers, we first evaluated the expression of USP15 in human pan-cancer using the Cancer Genome Atlas (TCGA) data provided by GEPIA (Gene Expression Profiling Interactive analysis, http://gepia.cancer-pku.cn/detail.php?gene=USP15). Among 33 different human cancer types based on molecular similarity (Fig. 1A), five different cancer types, lung adenocarcinoma (LUAD), lung squamous cell carcinoma (LUSC), ovarian serous cystadenocarcinoma (OV), skin cutaneous melanoma (SKCM), and testicular germ cell tumors (TGCT), showed significantly downregulated expression of USP15 (Fig. 1B, tumor (T) vs. normal (N)), whereas no significant change in expression of USP15 was observed in 28 other cancer types (Fig. 1A, black letters). The expression of USP15 was markedly lower in those with lung cancer than in normal controls (Fig. 1C, 27.29 in lung cancer vs. 64.76 in normal controls). In addition, the survival rate from combined patients with LUAD and LUSC decreased in patients with low expression levels of USP15 (Supplementary Fig. S1, blue line with low USP15 vs. red line with high USP15), indicating that USP15 expression may have an essential role in lung cancer patient’s survival. To verify the TCGA cancer data in those with primary lung cancer, we assessed the expression of USP15 in 41 patients with primary non-small cell lung cancer (NSCLC) [18, 19], including 25 patients with lung adenocarcinoma (LUAD, blue bars in Fig. 1D), 8 patients with lung squamous cell carcinoma (LUSC, red bars in Fig. 1D), and 8 patients with other lung cancers (green bars in Fig. 1D). LUAD patients showed significant downregulation of USP15 (Fig. 1D, blue bars). These findings suggest that USP15 might be functionally associated with lung adenocarcinoma. Therefore, we explored whether the expression of USP15 was implicated in the progression of human lung adenocarcinoma. To explore the functional role of USP15 in human lung adenocarcinoma, we generated *USP15*-knockout (*USP15*KO) human lung adenocarcinoma A549 and *USP15*KO human non-small cell lung cancer H1299 cells using CRISPR/Cas9 gene-editing method (Fig. 2A, *USP15*KO A549; Fig. 2B, *USP15*KO H1299) as described in “Materials and methods”. With *USP15*KO A549 and *USP15*KO H1299 lung cancer cells, we preliminary assessed whether *USP15* deficiency affected lung cancer invasion using a transwell invasion assay. The invasion of *USP15*KO A549 cells was significantly increased than that of control (Ctrl) A549 cells (Fig. 2C, D, *USP15*KO A549 vs. Ctrl A549 cells). Similarly, the invasion ability of *USP15*KO H1299 cells was also significantly higher than that of Ctrl H1299 cells (Fig. 2E, F, *USP15*KO H1299 vs. Ctrl H1299 cells), suggesting that USP15 might be functionally implicated in lung cancer invasion.

### *USP15*KO A549 lung cancer cells exhibit increased lung cancer progression induced by TLR4 stimulation through enhanced autophagy

**Fig. 3:**
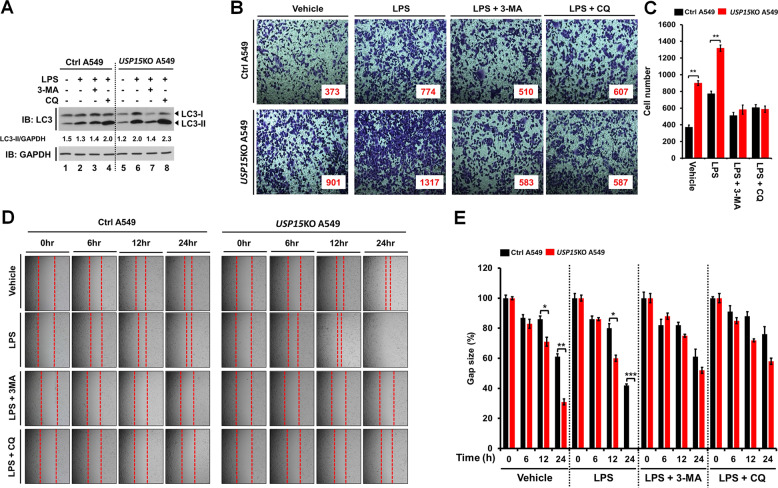
*USP15*KO A549 cells show increases in autophagy induction, migration, and invasion induced by TLR4 stimulation. **A** Control (Ctrl) and *USP*15KO A549 cells were treated with LPS, CQ, and 3-MA as indicated. Cell lysates were immunoblotted with antibodies specific for LC3-I/-II or GAPDH. LC3-II levels were analyzed with the Image J quantification tool. **B**, **C** Ctrl and *USP15*KO A549 cells were suspended in RPMI medium including vehicle, LPS (10 μg/mL), CQ (10 μM) or 3-MA (5 mM) plus LPS (10 μg/mL) and placed on top chambers of 24-transwell plates. After overnight incubation, cells were fixed and stained with crystal violet (**B**). The number of migrating cells was counted. Results are presented as mean ± SEM of three independent experiments (**C**). *P < 0.05; **P < 0.01. **D,** **E** Ctrl and *USP*15KO A549 cells were seeded into 12-well cell culture plates. Confluent monolayers were scraped with a sterile yellow Gilson-pipette tip. The wound was then treated with vehicle (DMSO, <0.2% in culture medium), LPS (10 μg/mL), CQ (10 μM) or 3-MA (5 mM) plus LPS (10 μg/mL) for different time periods as indicated. A representative experiment was shown (**D**). The residual gap between migrating cells from the opposing wound edge was expressed as a percentage of the initial scraped area (±SEM, *n* = 3) (**E**). *P < 0.05; **P < 0.01; and ***P < 0.001.

**Fig. 4:**
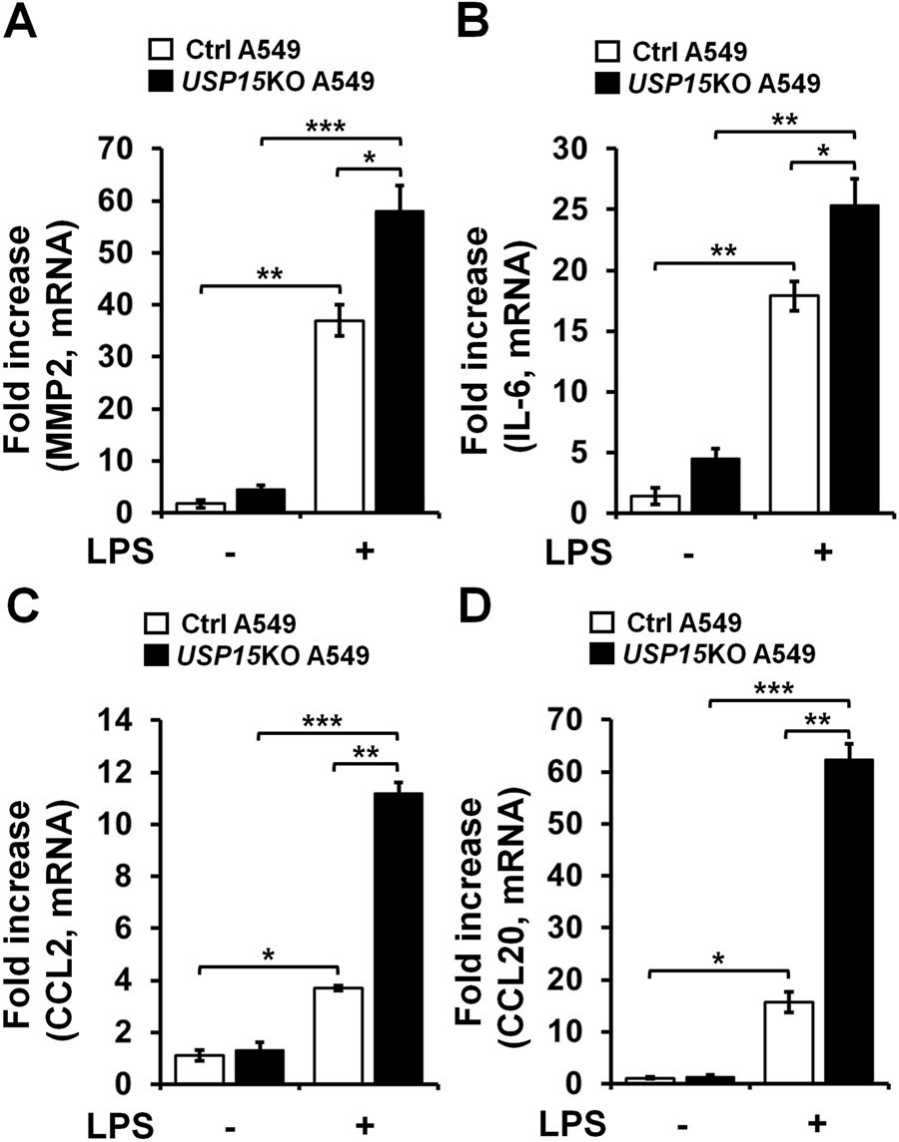
MMP2, IL-6, CCL2, and CCL20 mRNA levels are increased in *USP15*KO A549 cells in response to TLR4 stimulation. **A**–**D** Ctrl and *USP15*KO A549 cells were treated without or with 10 μg/mL LPS as indicated. Total RNA was extracted, cDNA was synthesized, and RT−qPCR analysis was performed with specific primers for MMP2 (**A**), IL-6 (**B**), CCL2 (**C**), and CCL20 (**D**). Results are presented as mean ± SEM of three independent experiments. *P < 0.05; **P < 0.01; and ***P < 0.001.

Previous reports have demonstrated that TLR3/4 activation can increase the production of IL-6, CCL2, MMP2, and CCL20 cytokines by promoting TRAF6 ubiquitination and autophagy induction, thereby facilitating the migration and invasion of lung cancer cells [7, 10, 11]. Having found that *USP15*KO A549 and *USP15*KO H1299 lung cancer cells exhibited increased cancer invasion, we further examined whether USP15 affected lung cancer migration and invasion through autophagy induction by TLR4 stimulation. The level of LC3-II specifically associated with autophagosomes and autolysosomes [28] was markedly enhanced in *USP15*KO A549 cells treated with LPS than in Ctrl A549 cells treated with LPS (Fig. 3A, *USP15*KO A549 with LPS vs. Ctrl A549 with LPS). In addition, the basal level of LC3-II/-I increased in *USP15*KO A549 cells without LPS (Supplementary Fig. S2, *USP15*KO A549 vs, Ctrl A549), indicating that the expression of USP15 affects the generation of LC3-II at the basal statue under the absence of LPS stimulation. As expected, autophagy inhibitors 3-MA (an inhibitor of phosphatidylinositol 3-kinases (PI3K) that can induce a decrease of LC3-II in cells) [29] and CQ (known to block the binding of autophagosomes to lysosomes and induce accumulation of LC3-II in cells) [30] induced a decrease and an increase of LC3-II in both cells treated with LPS, respectively (Fig. 3A, lane 2 vs. lane 3 or lane 4 in Ctrl A549 cells; lane 6 vs. lane 7 or lane 8 in *USP15*KO A549). Importantly, cancer invasion induced by TLR4 stimulation was markedly enhanced in *USP15*KO A549 cells treated with LPS, whereas it was inhibited by co-treatment of LPS with 3-MA or CQ (Fig. 3B, C, LPS vs. vehicle, or LPS + 3-MA and LPS + CQ vs. LPS in Ctrl A549 and *USP15*KO A549). Similar results were found for cancer migration (Fig. 3D, E, *USP15*KO A549 vs. Ctrl A549). Autophagy induced by TLR4 or TLR3 activation can enhance the production of cytokines such as IL-6, CCL2, MMP2, and CCL20 by promoting TRAF6 ubiquitination, thus facilitating the migration and invasion of lung cancer cells [7, 10, 11]. Therefore, we assessed whether USP15 was associated with the production of these cytokines induced by TLR4 stimulation. Interestingly, expression levels of MMP2, IL-6, CCL2, and CCL20 induced by TLR4 stimulation were significantly elevated in *USP15*KO A549 cells than in Ctrl A459 cells (Fig. 4A, MMP2; Fig. 4B, IL-6; Fig. 4C, CCL2; Fig. 4D, CCL20). These results suggest that USP15 is negatively implicated in the migration and invasion of lung cancer through the regulation of autophagy in response to TLR4 stimulation.

### *USP15*KO H1299 lung cancer cells exhibit increases in cancer migration and invasion induced by TLR4 stimulation

**Fig. 5:**
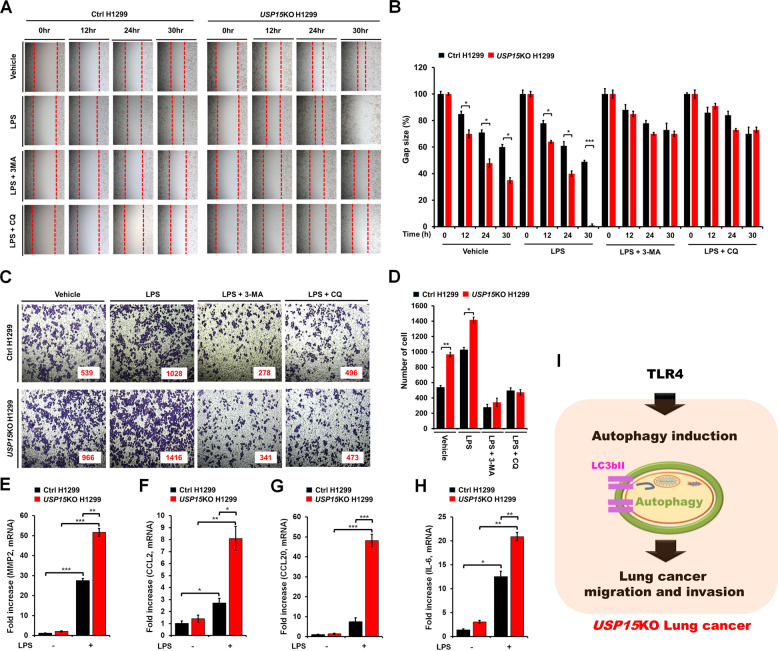
*USP15*KO H1299 cells exhibit increases in cell migration and invasion induced by TLR4 stimulation. **A**, **B** Control (Ctrl) and *USP*15KO H1299 cells were seeded into 12-well cell culture plates. Confluent monolayers were scraped with a sterile yellow Gilson-pipette tip. The wound was then treated with vehicle (DMSO, <0.2% in culture medium), LPS (10 μg/mL), CQ (10 μM) or 3-MA (5 mM) plus LPS (10 μg/mL) for different time periods as indicated. A representative experiment was shown (**A**). The residual gap between migrating cells from the opposing wound edge was expressed as a percentage of the initial scraped area (±SEM, *n* = 3) (**B**). *P < 0.05; ***P < 0.001. **C**, **D** Ctrl and *USP15*KO H1299 cells were suspended in RPMI medium including vehicle, LPS (10 μg/mL), CQ (10 μM) or 3-MA (5 mM) plus LPS (10 μg/mL) and placed on top chambers of 24-transwell plates. After overnight incubation, cells were fixed and stained with crystal violet (**C**). The number of migrating cells was counted. Results are presented as mean ± SEM of three independent experiments (**D**). *P < 0.05; **P < 0.01. **E**–**H** Ctrl and *USP15*KO H1299 cells were treated without or with 10 μg/mL LPS as indicated. Total RNA was extracted, cDNA was synthesized, and RT−qPCR analysis was performed with specific primers for *MMP2* (**E**), *CCL2* (**F**), *CCL20* (**G**), and *IL-6* (**H**). Results are presented as mean ± SEM of three independent experiments. *P < 0.05; **P < 0.01; ***P < 0.001. **I** A schematic model of the induction of autophagy induced by TLR4, thereby enhancing migration and invasion of *USP15*KO lung cancer cells.

Given the above results of *USP15*KO A549 cells, we verified the role of USP15 in *USP15*KO H1299 human non-small cell lung cancer cells. The migration of *USP15*KO H1299 cells was significantly enhanced in the presence or absence of LPS than that of Ctrl H1299 cells (Fig. 5A, B, *USP15*KO H1299 cells treated with vehicle or LPS vs. Ctrl H1299 cells treated with vehicle or LPS). Furthermore, cancer invasion was significantly higher in *USP15*KO H1299 cells treated with LPS than in Ctrl H1299 cells treated with LPS (Fig. 5C, D, *USP15*KO H1299 with LPS vs. Ctrl H1299 with LPS). Consistent with these results in *USP15*KO A549 cells (Fig. 3B–E), co-treatment of cells with LPS and 3-MA or CQ resulted in marked decreases of cancer migration and invasion induced by LPS (Fig. 5A, B, LPS vs. LPS + 3-MA or LPS + CQ; Fig. 5C, D, LPS vs. LPS + 3-MA or LPS + CQ). Moreover, expression levels of MMP2, CCL2, CCL20, and IL-6 induced by LPS stimulation were significantly elevated in *USP15*KO H1299 cells than in Ctrl H1299 cells (Fig. 5E, MMP2; Fig. 5F, CCL2; Fig. 5G, CCL20; Fig. 5H, IL-6 in *USP15*KO H1299 cells treated with LPS vs. Ctrl H1299 cells treated with LPS). These results strongly suggest that the deficiency of USP15 in lung cancer enhances cancer migration and invasion induced by TLR4 stimulation by increasing autophagy induction as depicted in Fig. 5I.

### USP15 interacts with BECN1 and induces deubiquitination of BECN1

**Fig. 6:**
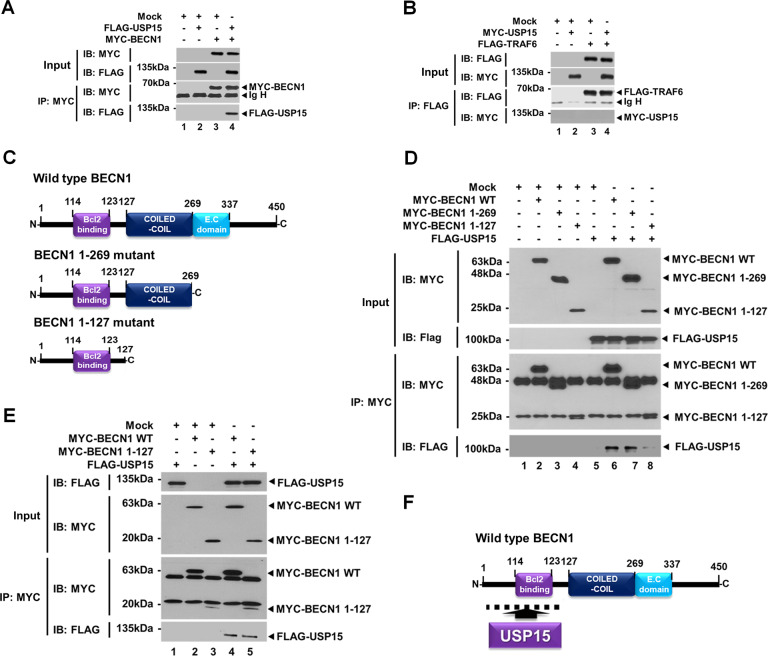
USP15 interacts with BECN1. **A** Immunoprecipitation (IP) assay was performed with anti-MYC antibody using HEK 293T cells transfected with mock, FLAG-USP15, and MYC-BECN1 as indicated. Immunoblotting (IB) assay was performed with anti-FLAG or anti-MYC antibody. **B** IP assay was performed with anti-FLAG antibody using HEK 293 T cells transfected with mock, MYC-USP15, and FLAG-TRAF6 as indicated. IB assay was performed with anti-FLAG or anti-MYC antibody. **C** Truncated mutants of BECN1, BECN1 1-269, and BECN1 1-127 mutants were generated as described in “Materials and methods”. **D** IP assay was performed with anti-MYC antibody using HEK 293T cells transfected with mock, MYC-BECN1 wild-type (WT), MYC-BECN1 1-269, MYC-BECN1 1-127, and FLAG-USP15 as indicated. IB assay was performed with anti-FLAG or anti-MYC antibodies. **E** IP assay was performed with anti-MYC antibody using HEK 293T cells transfected with mock, MYC-BECN1 wild-type WT, MYC-BECN1 1-127, and FLAG-USP15, as indicated. IB assay was performed with anti-FLAG or anti-MYC antibody. **F** A schematic model for the interaction between BECN1 and USP15.

Having shown that USP15 negatively regulated lung cancer migration and invasion induced by TLR4 stimulation through inhibition of autophagy induction, we next explored the molecular mechanism in which USP15 was implicated in autophagy induction by TLR4 stimulation. Ubiquitination of BECN1 by TRAF6 plays a pivotal role in autophagy induction in response to TLR4 stimulation [7, 10, 11]. In addition, we have recently reported that USP15 can dampen NF-κB activation through deubiquitination of IκBα [16]. Therefore, the regulatory mechanism of USP15 in autophagy induction might be functionally associated with the ubiquitination of BECN1. Interestingly, USP15 interacted with BECN1 (Fig. 6A, lane 4), whereas no significant interaction was observed between USP15 and TRAF6 (Fig. 6B, lane 4). To elucidate the molecular association between USP15 and BECN1, BECN1 truncated mutants were generated (Fig. 6C), and immunoprecipitation (IP) assay was performed with USP15. FLAG-USP15 interacted with MYC-BECN1 wild-type (WT) and MYC-BECN1 1-269 mutant (Fig. 6D, lanes 6 and 7), whereas it showed a weak interaction with MYC-BECN1 1-127 (Fig. 6D, lane 8). To confirm the interaction between USP15 and BECN1 1-127 mutant, IP assay was further performed. As shown in Fig. 6E, FLAG-USP15 interacted with MYC-BECN1 WT and MYC-BECN1 1-127 mutant (Fig. 6E, lanes 4 and 5), indicating that USP15 could interact with the N-terminal domain of BECN1 (Fig. 6F).

**Fig. 7:**
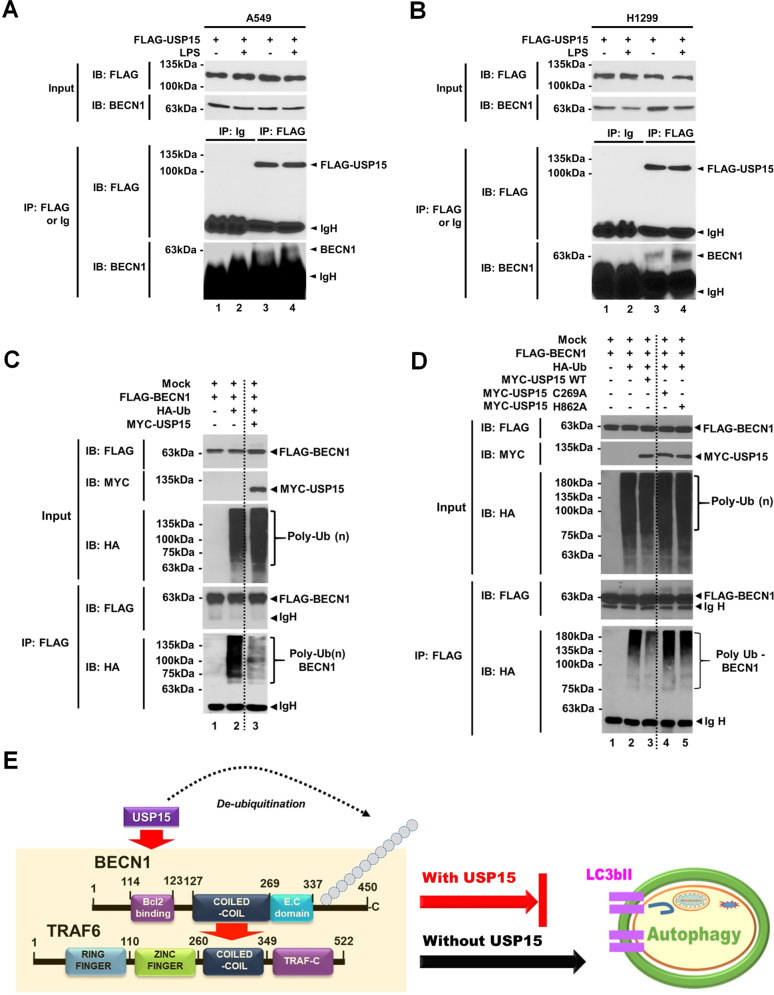
USP15 induces deubiquitination of BECN1. **A**, **B** A549 (**A**) and H1299 (**B**) lung cancer cells were transiently transfected with FLAG-USP15. Immunoprecipitation (IP) assay was performed with anti-Ig or anti-FLAG antibodies, and then immune-probed with anti-FALG and anti-BECN1 antibodies, as indicated. **C** Immunoprecipitation (IP) assay was performed with anti-FLAG antibody using HEK 293T cells transfected with mock, FLAG-BECN1, HA-UB, and MYC-USP15 as indicated. Immunoblotting (IB) assay was performed with anti-FLAG or anti-HA antibody. **D** IP assay was performed with anti-FLAG antibody using HEK 293T cells transfected with mock, FLAG-BECN1, HA-Ub, MYC-USP15 wild-type (WT), MYC-USP15 C269A, and MYC-USP15 H862A as indicated. IB assay was performed with anti-FLAG or anti-HA antibody. **E** A schematic model of how USP15 induces deubiquitination of BECN1. In the presence of USP15, UPS15 interacts with BECN1 and induces deubiquitination of BECN1, leading to the inhibition of autophagy (indicated as a red arrow). In the absence of USP15, in contrast, TRAF6 interacts with BECN1 and induces ubiquitination of BECN1, leading to the induction of autophagy (indicated as a black arrow).

To further verify the molecular interaction between USP15 and BECN1, we performed a semi-endogenous IP assay because the anti-USP15 antibody used in this study was not suitable for IP assay. FLAG-USP15 was transiently expressed in A549 and H1299 lung cancer cells, and then the interaction with endogenous BECN1 was evaluated (Fig. 7A, A549; Fig. 7B, H1299). FLAG-USP15 interacted with endogenous BECN1 in the absence of LPS stimulation, and the interaction was significantly enhanced in the presence of LPS stimulation (Fig. 7A, B, lane 4 vs. lane 3), indicating that USP15 interacts with endogenous BECN1. Based on the molecular association between USP15 and BECN1, we asked whether USP15 could induce deubiquitination of BECN1. FLAG-BECN1 and HA-Ub were transfected in the presence or absence of MYC-USP15. The ubiquitination of BECN1 appeared in the absence of MYC-USP15 (Fig. 7C, lane 2 vs. lane 1), whereas it was markedly attenuated in the presence of MYC-USP15 (Fig. 7C, lane 3 vs. lane 2). To verify whether the deubiquitination of BECN1 was dependent on the catalytic activity of USP15, we generated two catalytic mutants of USP15, USP15 C269A, and USP15 H862A [25]. A ubiquitination assay was then performed. Consistently, the wild-type USP15 induced deubiquitination of BECN1 (Fig. 7D, lane 3 vs. 2), whereas the two catalytic mutants of USP15 failed to induce deubiquitination of BECN1 (Fig. 7D, lanes 4 and 5 vs. 3), indicating that the deubiquitination of BECN1 was dependent on the catalytic activity of USP15. As depicted in Fig. 7E, these results suggest that the molecular association of TRAF6-BECN1 can lead to ubiquitination of BECN1 and induce autophagy induction, whereas the interaction of USP15 with BECN1 can induce the deubiquitination of BECN1 and inhibit autophagy induction.

### Downregulation of USP15 is associated with genes related to lung cancer progression in patients with primary non-small cell lung cancer (NSCLC)

**Fig. 8:**
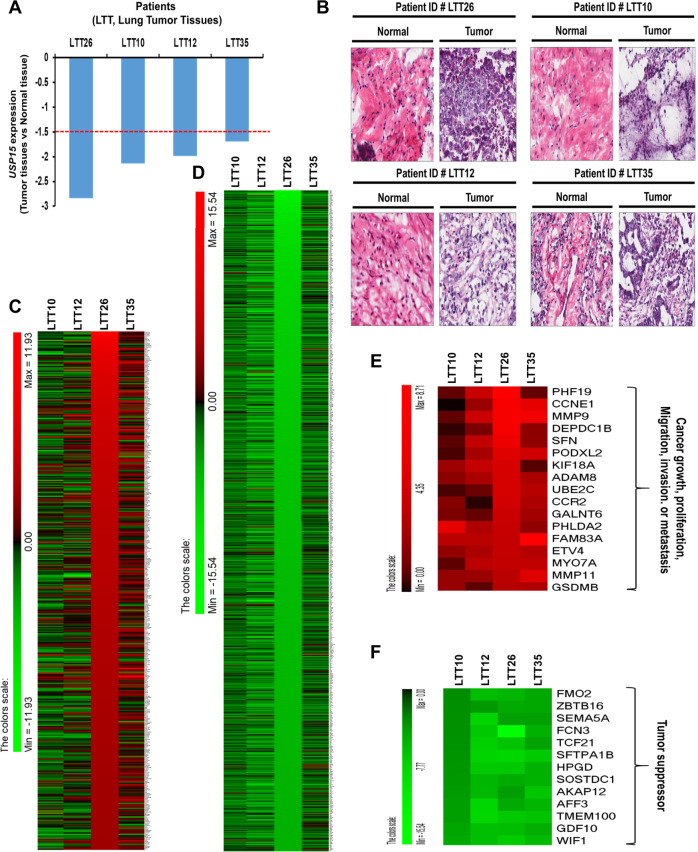
NSCLC patients with low expression levels of USP15 exhibit upregulated expression levels of genes related to lung cancer progression. **A** Among 41 patients with primary NSCLC, four patients with NSCLC (LTT26, LTT10, LTT12, and LTT35) with low expression of USP15 were selected and their relative expression levels of USP15 were presented. **B** In primary lung tumor and matched normal tissues, H&E (hematoxylin and eosin) staining was performed as described in “Materials and methods”. **C**, **D** From tumor and matched normal tissues of LTT10, LTT12, LTT26, and LTT35 patients with NSCLC, microarray analysis was performed as described in “Materials and methods”. Based on the results of LTT26 patient with the lowest expression of USP15 among 41 primary NSCLC patients, upregulated genes (**C**) and downregulated genes (**D**) were sorted out and presented. **E** Among upregulated genes presented in (**C**), 17 different cancer-associated genes commonly upregulated in four primary NSCLC patients were sorted out and presented. **F** Among downregulated genes presented in (**D**), 13 different tumor suppressor genes commonly upregulated in four primary NSCLC patients were sorted out and presented.

Given the results that USP15 negatively regulated lung cancer migration and invasion through inhibition of autophagy induction by deubiquitination of BECN1, we tried to find the clinical relevance of USP15 in the regulation of lung cancer progression. To do that, four patients with primary adenocarcinoma who showed significantly downregulated USP15 (< −1.5 values in the relative expression of *USP15* in tumor and matched normal tissues) were selected (Fig. 8A, LTT26, LTT10, LTT12, and LTT35) among 41 patients with primary non-small cell lung cancer (NSCLC) (Fig. 1D). H&E staining presented significant pathological differences between matched normal and lung tumor tissues in terms of the structure of lung tissue and the number of cancer cells (Fig. 8B, normal vs. tumor). Given these results, microarray analysis was performed for matched normal and lung tumor tissues using Human HT-12 expression BeadChips. To select upregulated and/or downregulated genes in four LTTs, LTT26 tumor patient with the lowest expression of USP15 was used as a standard sample (Fig. 8C and Supplementary Table S1, upregulated genes; Fig. 8D and Supplementary Table S2, downregulated genes). Among 17 commonly upregulated genes in four LTT patients (Fig. 8E and Supplementary Table S3), 15 genes were functionally associated with lung cancer growth, proliferation, migration, invasion, or metastasis: 10 genes (*CCNE1* [31], *MMP9* [32], *SFN* [33], *UBE2C* [34], *CCR2* [35], *FAM83A* [36], *ETV4* [37], *MYO7A* [38], *MMP11* [39], and *GSDMB* [40]) can promote lung cancer progression, two genes (*DEPDC1B* [41] and *GALNT6* [42]) can promote lung cancer migration and invasion, and three genes (*KIF18A* [43], *ADAM8* [44], and *PHLDA2* [45]) can promote lung cancer proliferation, metastasis, or oncogenesis. Among 13 commonly downregulated genes in four patients (Fig. 8F and Supplementary Table S4), 10 genes (*FMO2* [45], *ZBTB16* [46], *FCN3* [47], *TCF21* [48], *SFTPA1B* [49], *HPGD* [50], *SOSTDC1* [51], *TMEM100* [52], *GDF10* [53], and *WIF1* [54]) have been reported as tumor suppressors in lung cancer. These results suggest that downregulation of USP15 in lung cancer might be associated with gene expression capable of affecting lung cancer progression and formation.

## Discussion

This study demonstrates that USP15 is negatively implicated in lung cancer progression through the regulation of TRAF6-BECN1 signaling for autophagy induction for the first time. Importantly, TCGA data analysis revealed that USP15 was significantly downregulated in lung adenocarcinoma (LUAD) and lung squamous cell carcinoma (LUSC). Consistently, the expression of USP15 was significantly downregulated in patients with primary lung adenocarcinoma. Notably, the profound impact of USP15 expression was shown in *USP15*KO A549 and *USP15*KO H1299 lung cancer cells. *USP15*KO lung cancer cells exhibited increased cancer migration and invasion induced by TLR4 stimulation through the regulation of autophagy. Biochemical studies have revealed that USP15 can induce the deubiquitination of BECN1, a key regulator of the induction of autophagy [7, 10–12]. More importantly, microarray analysis in four patients with primary lung adenocarcinoma who had low expression of USP15, revealed that 10 different genes (*CCNE1, MMP9, SFN, UBE2C, CCR2, FAM83A, ETV4, MYO7A, MMP11,* and *GSDMB*) that are associated with promoting lung cancer progression were significantly upregulated, whereas 10 different genes (*FMO2, ZBTB16, FCN3, TCF21, SFTPA1B, HPGD, SOSTDC1, TMEM100, GDF10*, and *WIF1*) known to suppress lung cancer were markedly downregulated.

TRAF6 signals are known to play critical roles in differential biological contexts including innate and adaptive immunity and tumor development [8]. A distinct TRAF6-mediated signaling pathway has been revealed through its seemingly innumerable interaction partners and its E3 ubiquitin ligase activity [8]. Recent studies have shown that the TRAF6-BECN1 signaling axis plays a key role in the induction of autophagy, thereby promoting lung cancer migration and invasion in response to TLR4 stimulation [7, 10–12]. Importantly, autophagy functionally facilitates TLR4-induced lung cancer progression by enhancing TRAF6-mediated ubiquitination of BECN1 and the production of IL-6, CCL2/MCP-1, CCL20/MIP-3α, VEGFA, and MMP2 [7, 10–12], strongly suggesting a pivotal role of the TRAF6-BECN1 signaling axis in lung cancer progression. In the current work, we found that USP15 interacted with BECN1, but not with TRAF6. The interaction between USP15 and BECN1 led to the deubiquitination of BECN1, resulting in the attenuation of autophagy induction. Similarly, we have previously reported that USP15 can inhibit NF-κB activation induced by RANK signaling through deubiquitination of IκB-α [16], supporting that USP15 is a deubiquitinating enzyme that is functionally implicated in various cellular contexts.

Regarding the cellular function of USP15 in lung cancer progression, notably, *USP15*KO A549 and *USP15*KO H1299 lung cancer cells exhibited increases in cancer invasion that were markedly enhanced in response to TLR4 stimulation after treatment with LPS. In contrast, co-treatment of these cells with LPS and autophagy inhibitors such as 3-MA and CQ attenuated cancer migration and invasion induced by TLR4 stimulation. Based on the molecular mechanism by which USP15 is negatively implicated in the autophagy induction through deubiquitination of BECN1, our study suggests that the negative regulation of USP15 in lung cancer progression might be functionally implicated in the regulation of autophagy induction. In terms of clinical aspects, we found that the expression of USP15 was significantly downregulated in those with LUAD and LUSC. Consistently, USP15 was significantly downregulated in patients with primary lung adenocarcinoma, strongly suggesting that USP15 might be functionally associated with lung cancer. Importantly, patients with low expression levels of USP15 showed increased expression of 17 different genes related to cancer progression and formation and decreased expression of 13 different genes known to suppress tumors. Among these 17 different genes, 10 genes (*CCNE1, MMP9, SFN, UBE2C, CCR2, FAM83A, ETV4, MYO7A, MMP11*, and *GSDMB*) are known to promote lung cancer progression and formation. Among the 13 different genes known to suppress tumors, 10 genes (*FMO2, ZBTB16, FCN3, TCF21, SFTPA1B, HPGD, SOSTDC1, TMEM100, GDF10*, and *WIF1*) are known to suppress lung cancer. These results suggest that USP15 is negatively implicated in the progression and formation of lung cancer.

**Fig. 9:**
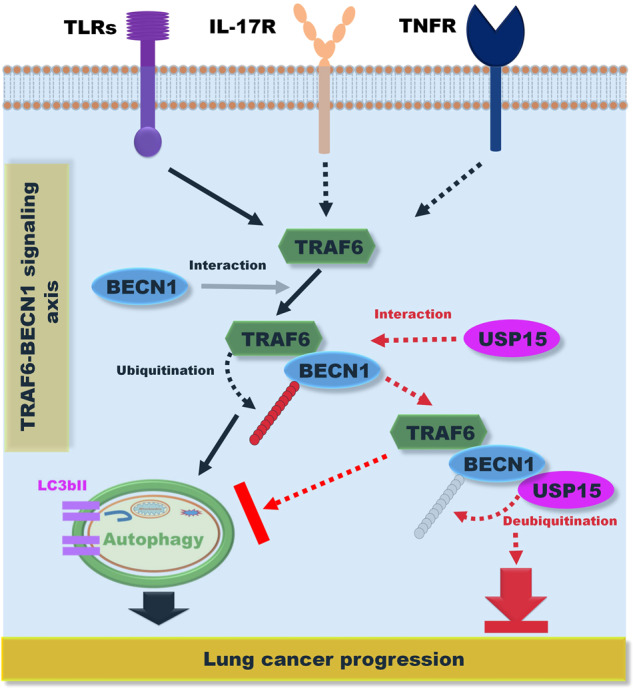
A schematic model showing how USP15 regulates lung cancer progression by regulating the TRAF6-BECN1 signaling axis for the induction of autophagy. Upon cellular stimuli including TLRs, IL-17R, and TNFR, TRAF6 functionally regulates the occurrence and development of tumors through various cellular events, such as cell apoptosis, proliferation, survival, and invasion. Regarding the TRAF6-BECN1 signaling axis for cancer progression (depicted in the left), TRAF6 can interact with BECN1 and induce ubiquitination of BECN1, leading to the induction of autophagy (indicated as a black arrow). UPS15 can interact with BECN1 and induce deubiquitination of BCEN1, thereby inhibiting the induction of autophagy (indicated as red dashed arrow). Eventually, USP15 is negatively implicated in lung cancer progression by inhibiting the TRAF6-BECN1 signaling axis.

Autophagy plays a dual role in cancer development by suppressing the growth of tumors or cancer progression [55, 56]. The upregulation of autophagy leads to the resistance of cancer cells to various anticancer drugs [56, 57]. Upon cellular stimuli including TLRs, IL-17R, and TNFR, TRAF6 regulates tumor cell proliferation, survival, apoptosis, and invasion through different signaling pathways [8, 58]. As depicted in Fig. 9, TRAF6 has oncogenic characteristics involved in cancer progression. Importantly, recent studies have reported that autophagy facilitates TLR-induced migration and invasion of lung cancer cells through the TRAF6-induced ubiquitination of BECN1 [7, 10–12], suggesting that TLR-induced autophagy is functionally associated with lung cancer progression. On the other hand, accumulated evidence has proven that TLRs play a critical role in inducing anti-tumor effects by eliciting inflammatory cytokine expressions and innate immune response through the activation of NF-κB [59]. Although TLRs are essential for the immune response against tumor cells, recent studies have demonstrated that TLRs are expressed on tumor cells and crucially functioned in tumorigenesis and tumor progression through their downstream signaling pathways [7, 10–12, 59]. Indeed, TLRs act as critical sensors that regulate lung cancer progression including cell growth and invasion and migration and metastasis through the activation of NF-κB and autophagy [7, 10–12]. Although the precise roles and fundamental mechanisms by which TLR-induced autophagy is implicated in lung cancer progression need to be further elucidated, the pathway of TLR-induced autophagy can be potentially considered as a therapeutic target for alleviating lung cancer progression.

In summary, we proposed a possible mechanism by which USP15 regulates TRAF6-BECN1 signaling in autophagy induction and lung cancer progression. As depicted in Fig. 9, regarding the TRAF6-BECN1 signaling axis for cancer progression in response to TLR4 stimulation (depicted in the left), TRAF6 can interact with BECN1 and induce ubiquitination of BECN1, leading to the induction of autophagy (indicated as a black arrow). USP15 can interact with BECN1 and induce deubiquitination of BCEN1, thereby inhibiting the induction of autophagy (indicated as red dashed arrow). Eventually, USP15 can negatively regulate lung cancer progression by inhibiting the TRAF6-BECN1 signaling axis through autophagy induction. Although significant progress has been achieved in exploring the roles of USPs in cancers, very little is known about the molecular and cellular mechanism by which USPs are implicated in lung cancer progression and formation. Our current results provide insight into pathological lung cancer processes and the future development of therapeutic agents for treating lung cancer.

## Supplementary information

Supplementary information

Supplemental Material

Checklist

Supplementary Table S1

Supplementary Table S2

Supplementary Table S3

Supplementary Table S4

## Data Availability

The data that support the findings of this study are available from the corresponding author upon reasonable request.

## References

[1] Mathew R, Karantza-Wadsworth V, White E (2007). Role of autophagy in cancer. Nat Rev Cancer.

[2] Bhutia SK, Mukhopadhyay S, Sinha N, Das DN, Panda PK, Patra SK (2013). Autophagy: cancer’s friend or foe?. Adv Cancer Res.

[3] Yim WW, Mizushima N (2020). Lysosome biology in autophagy. Cell Discov.

[4] Nakamura S, Yoshimori T (2017). New insights into autophagosome-lysosome fusion. J Cell Sci.

[5] Glick D, Barth S, Macleod KF (2010). Autophagy: cellular and molecular mechanisms. J Pathol.

[6] Kelekar A, Ann NY (2005). Autophagy. Acad Sci.

[7] Zhan Z, Xie X, Cao H, Zhou X, Zhang XD, Fan H (2014). Autophagy facilitates TLR4- and TLR3-triggered migration and invasion of lung cancer cells through the promotion of TRAF6 ubiquitination. Autophagy..

[8] Walsh MC, Lee J, Choi Y (2015). Tumor necrosis factor receptor-associated factor 6 (TRAF6) regulation of development, function, and homeostasis of the immune system. Immunol Rev.

[9] Yin Z, Popelka H, Lei Y, Yang Y, Klionsky DJ (2020). The roles of ubiquitin in mediating autophagy. Cells..

[10] Min Y, Kim MJ, Lee S, Chun E, Lee KY (2018). Inhibition of TRAF6 ubiquitin-ligase activity by PRDX1 leads to inhibition of NFKB activation and autophagy activation. Autophagy..

[11] Kim MJ, Min Y, Im JS, Son J, Lee JS, Lee KY (2020). p62 is negatively implicated in the TRAF6-BECN1 signaling axis for autophagy activation and cancer progression by toll-like receptor 4 (TLR4). Cells..

[12] Kim MJ, Min Y, Shim JH, Chun E, Lee KY (2019). CRBN is a negative regulator of bactericidal activity and autophagy activation through inhibiting the ubiquitination of ECSIT and BECN1. Front Immunol.

[13] Reyes-Turcu FE, Ventii KH, Wilkinson KD (2009). Regulation and cellular roles of ubiquitin-specific deubiquitinating enzymes. Annu Rev Biochem.

[14] Chou CK, Chang YT, Korinek M, Chen YT, Yang YT, Leu S (2017). The regulations of deubiquitinase USP15 and its pathophysiological mechanisms in diseases. Int J Mol Sci.

[15] Teyra J, Singer AU, Schmitges FW, Jaynes P, Kit Leng Lui S, Polyak MJ (2019). Structural and functional characterization of ubiquitin variant inhibitors of USP15. Structure..

[16] Greenblatt MB, Park KH, Oh H, Kim JM, Shin DY, Lee JM (2015). CHMP5 controls bone turnover rates by dampening NF-κB activity in osteoclasts. J Exp Med.

[17] Cornelissen T, Haddad D, Wauters F, Van Humbeeck C, Mandemakers W, Koentjoro B (2014). The deubiquitinase USP15 antagonizes Parkin-mediated mitochondrial ubiquitination and mitophagy. Hum Mol Genet.

[18] Kim MJ, Min Y, Son J, Kim JY, Lee JS, Kim DH (2020). AMPKα1 regulates lung and breast cancer progression by regulating TLR4-mediated TRAF6-BECN1 signaling axis. Cancers..

[19] Kim Y, Lee BB, Kim D, Um S, Cho EY, Han J (2020). Clinicopathological significance of RUNX1 in non-small cell lung cancer. J Clin Med.

[20] Min Y, Lee S, Kim MJ, Chun E, Lee KY (2017). Ubiquitin-specific protease 14 negatively regulates toll-like receptor 4-mediated signaling and autophagy induction by inhibiting ubiquitination of TAK1-binding protein 2 and beclin 1. Front Immunol.

[21] Mi WiS, Park J, Shim JH, Chun E, Lee KY (2015). Ubiquitination of ECSIT is crucial for the activation of p65/p50 NF-κBs in Toll-like receptor 4 signaling. Mol Biol Cell.

[22] Wi SM, Moon G, Kim J, Kim ST, Shim JH, Chun E (2014). TAK1-ECSIT-TRAF6 complex plays a key role in the TLR4 signal to activate NF-κB. J Biol Chem.

[23] Kim MJ, Min Y, Kwon J, Son J, Im JS, Shin J (2019). p62 negatively regulates TLR4 signaling via functional regulation of the TRAF6-ECSIT complex. Immune Netw.

[24] Zhang H, Wang D, Zhong H, Luo R, Shang M, Liu D (2015). Ubiquitin-specific protease 15 negatively regulates virus-induced type I interferon signaling via catalytically-dependent and -independent mechanisms. Sci Rep..

[25] Min Y, Wi SM, Shin D, Chun E, Lee KY (2017). Peroxiredoxin-6 negatively regulates bactericidal activity and NF-κB activity by interrupting TRAF6-ECSIT complex. Front Cell Infect Microbiol.

[26] Kim SY, Jeong S, Jung E, Baik KH, Chang MH, Kim SA (2012). AMP-activated protein kinase-α1 as an activating kinase of TGF-β-activated kinase 1 has a key role in inflammatory signals. Cell Death Dis.

[27] Min Y, Wi SM, Kang JA, Yang T, Park CS, Park SG (2016). Cereblon negatively regulates TLR4 signaling through the attenuation of ubiquitination of TRAF6. Cell Death Dis.

[28] Runwal G, Stamatakou E, Siddiqi FH, Puri C, Zhu Y, Rubinsztein DC (2019). LC3-positive structures are prominent in autophagy-deficient cells. Sci Rep..

[29] Wu YT, Tan HL, Shui G, Bauvy C, Huang Q, Wenk MR (2010). Dual role of 3-methyladenine in modulation of autophagy via different temporal patterns of inhibition on class I and III phosphoinositide 3-kinase. J Biol Chem.

[30] Mauthe M, Orhon I, Rocchi C, Zhou X, Luhr M, Hijlkema KJ (2018). Chloroquine inhibits autophagic flux by decreasing autophagosome-lysosome fusion. Autophagy.

[31] Zhang Y, Shan C, Chen Y, Sun S, Liu D, Zhang X (2020). CircDENND2A promotes non-small cell lung cancer progression via regulating MiR-34a/CCNE1 signaling. Front Genet.

[32] El-Badrawy MK, Yousef AM, Shaalan D, Elsamanoudy AZ (2014). Matrix metalloproteinase-9 expression in lung cancer patients and its relation to serum mmp-9 activity, pathologic type, and prognosis. J Bronchol Inter Pulmonol.

[33] Shiba-Ishii A, Kim Y, Shiozawa T, Iyama S, Satomi K, Kano J (2015). Stratifin accelerates progression of lung adenocarcinoma at an early stage. Mol Cancer.

[34] Guo J, Wu Y, Du J, Yang L, Chen W, Gong K (2018). Deregulation of UBE2C-mediated autophagy repression aggravates NSCLC progression. Oncogenesis..

[35] An J, Xue Y, Long M, Zhang G, Zhang J, Su H (2017). Targeting CCR2 with its antagonist suppresses viability, motility and invasion by downregulating MMP-9 expression in non-small cell lung cancer cells. Oncotarget..

[36] Zheng YW, Li ZH, Lei L, Liu CC, Wang Z, Fei LR (2020). FAM83A promotes lung cancer progression by regulating the Wnt and Hippo signaling pathways and indicates poor prognosis. Front Oncol.

[37] Wang Y, Ding X, Liu B, Li M, Chang Y, Shen H (2020). ETV4 overexpression promotes progression of non-small cell lung cancer by upregulating PXN and MMP1 transcriptionally. Mol Carcinog.

[38] Liu Y, Wei X, Guan L, Xu S, Yuan Y, Lv D (2018). Unconventional myosin VIIA promotes melanoma progression. J Cell Sci.

[39] Yang H, Jiang P, Liu D, Wang HQ, Deng Q, Niu X (2019). Matrix metalloproteinase 11 is a potential therapeutic target in lung adenocarcinoma. Mol Ther Oncolytics.

[40] Xia X, Wang X, Cheng Z, Qin W, Lei L, Jiang J (2019). The role of pyroptosis in cancer: pro-cancer or pro-“host”?. Cell Death Dis.

[41] Yang Y, Liu L, Cai J, Wu J, Guan H, Zhu X (2014). DEPDC1B enhances migration and invasion of non-small cell lung cancer cells via activating Wnt/β-catenin signaling. Biochem Biophys Res Commun.

[42] Song J, Liu W, Wang J, Hao J, Wang Y, You X (2020). GALNT6 promotes invasion and metastasis of human lung adenocarcinoma cells through O-glycosylating chaperone protein GRP78. Cell Death Dis.

[43] Li X, Liu M, Zhang Z, Zhang L, Liang X, Sun L (2019). High kinesin family member 18A expression correlates with poor prognosis in primary lung adenocarcinoma. Thorac Cancer.

[44] Hernández I, Moreno JL, Zandueta C, Montuenga L, Lecanda F (2010). Novel alternatively spliced ADAM8 isoforms contribute to the aggressive bone metastatic phenotype of lung cancer. Oncogene..

[45] Hsu YL, Hung JY, Lee YL, Chen FW, Chang KF, Chang WA (2017). Identification of novel gene expression signature in lung adenocarcinoma by using next-generation sequencing data and bioinformatics analysis. Oncotarget..

[46] Xiao GQ, Li F, Unger PD, Katerji H, Yang Q, McMahon L (2016). ZBTB16: a novel sensitive and specific biomarker for yolk sac tumor. Mod Pathol.

[47] Jang H, Jun Y, Kim S, Kim E, Jung Y, Park BJ (2021). FCN3 functions as a tumor suppressor of lung adenocarcinoma through induction of endoplasmic reticulum stress. Cell Death Dis.

[48] Hu S, Yang N, Chen M, Guo J, Xian L. Effects of tumor suppressor gene TCF21 on the proliferation, migration and apoptosis of A549 cells. Zhongguo Fei Ai Za Zhi. 2014;17:302–7. 10.3779/j.issn.1009-3419.2014.04.03 PMC6000019 24758904

[49] Mitsuhashi A, Goto H, Kuramoto T, Tabata S, Yukishige S, Abe S (2013). Surfactant protein A suppresses lung cancer progression by regulating the polarization of tumor-associated macrophages. Am J Pathol.

[50] Ding Y, Tong M, Liu S, Moscow JA, Tai HH (2005). NAD+-linked 15-hydroxyprostaglandin dehydrogenase (15-PGDH) behaves as a tumor suppressor in lung cancer. Carcinogenesis..

[51] Chen G, Gong H, Wang T, Wang J, Han Z, Bai G (2018). SOSTDC1 inhibits bone metastasis in non-small cell lung cancer and may serve as a clinical therapeutic target. Int J Mol Med.

[52] Han Z, Wang T, Han S, Chen Y, Chen T, Jia Q (2017). Low-expression of TMEM100 is associated with poor prognosis in non-small-cell lung cancer. Am J Transl Res.

[53] Tandon M, Gokul K, Ali SA, Chen Z, Lian J, Stein GS (2012). Runx2 mediates epigenetic silencing of the bone morphogenetic protein-3B (BMP-3B/GDF10) in lung cancer cells. Mol Cancer.

[54] Chan SL, Cui Y, van Hasselt A, Li H, Srivastava G, Jin H (2007). The tumor suppressor Wnt inhibitory factor 1 is frequently methylated in nasopharyngeal and esophageal carcinomas. Lab Invest.

[55] Shin DW (2020). Dual roles of autophagy and their potential drugs for improving cancer therapeutics. Biomol Ther.

[56] Yun CW, Lee SH (2018). The roles of autophagy in cancer. Int J Mol Sci.

[57] Xiao X, Wang W, Li Y, Yang D, Li X, Shen C (2018). HSP90AA1-mediated autophagy promotes drug resistance in osteosarcoma. J Exp Clin Cancer Res.

[58] Wang J, Wu X, Jiang M, Tai G (2020). Mechanism by which TRAF6 participates in the immune regulation of autoimmune diseases and cancer. Biomed Res Int.

[59] Gu J, Liu Y, Xie B, Ye P, Huang J, Lu Z (2018). Roles of toll-like receptors: from inflammation to lung cancer progression. Biomed Rep..

