# Supplementary material for: USP15 negatively regulates lung cancer progression through the TRAF6-BECN1 signaling axis for autophagy induction
Source: Cell Death Dis. 2022 Apr 14;13(4):348. doi: 10.1038/s41419-022-04808-7 (PMC9010460; doi:10.1038/s41419-022-04808-7)
Supplement: Supplementary file 1 — Supplementary information [file 41419_2022_4808_MOESM1_ESM.docx]

**Supplement Figure Legends**

**
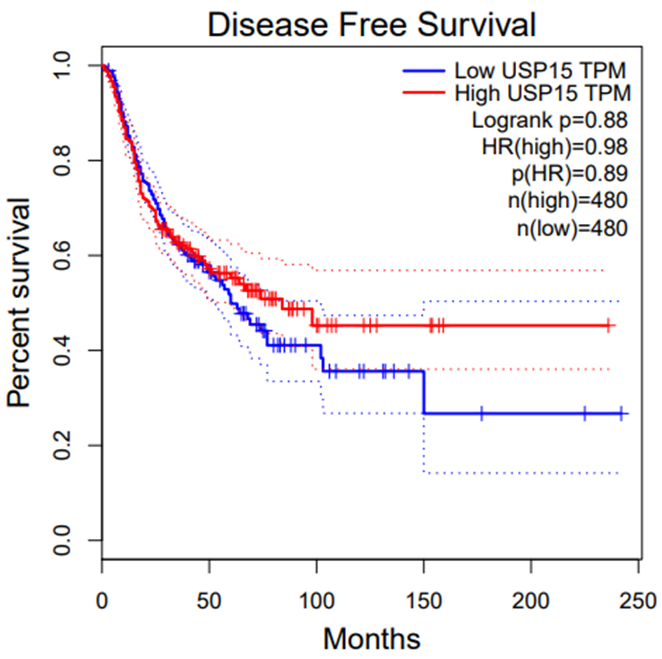
**

**Supplementary Figure S1. The percent survival of lung cancer patients with low expression of USP15 is decreased.**

The lung adenocarcinoma (LUAD) and lung squamous cell carcinoma (LUSC) combined Kaplan-Meier disease free survival analysis was performed using GAPIA TCGA data (<http://gepia.cancer-pku.cn/detail.php?gene=USP15>).

**
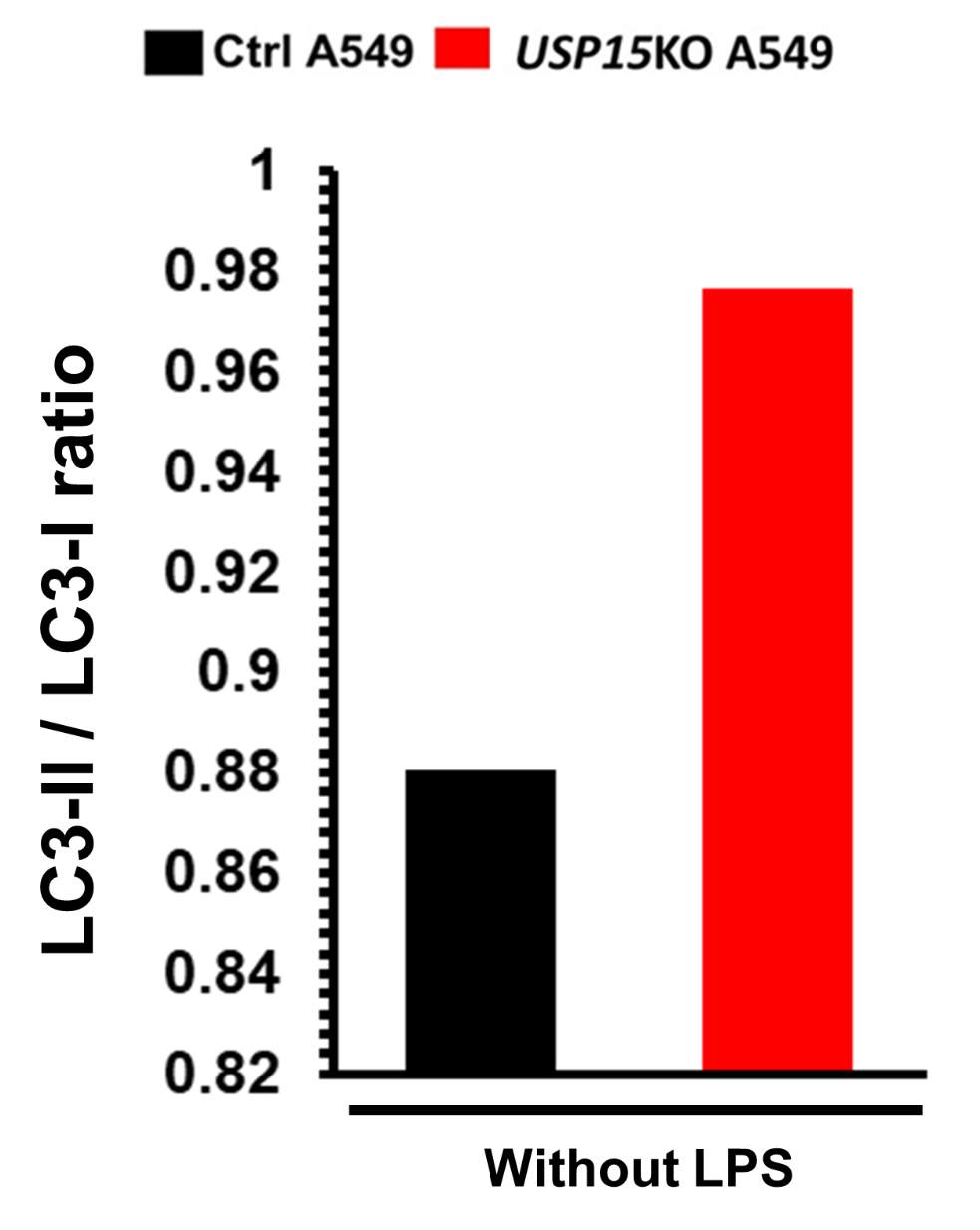
**

**Supplementary Figure S2. The basal level of LC3-II is increased in *USP15*KO A549 cells.** In Figure 3A, the basal level of LC3-II /LC3-1 between A549 and USP15KO A549 cells in the absence of LPS was analyzed with Image J quantification tool.

**Supplement Table information**

**Supplementary Table 1.** Up-regulated genes in LTT26 tumor patient are combined with those of LTT10, LTT12, and LTT35 tumor patients.

**Supplementary Table 2.** Down-regulated genes in LTT26 tumor patient are combined with those of LTT10, LTT12, and LTT35 tumor patients.

**Supplementary Table 3.** The list of 17 commonly up-regulated genes in four LTT tumor tissues, LTT10, LTT12, LTT26, and LTT35.

**Supplementary Table 4.** The list of 13 commonly down-regulated genes in four LTT tumor tissues, LTT10, LTT12, LTT26, and LTT35.
