# Supplementary material for: USP15 negatively regulates lung cancer progression through the TRAF6-BECN1 signaling axis for autophagy induction
Source: Cell Death Dis. 2022 Apr 14;13(4):348. doi: 10.1038/s41419-022-04808-7 (PMC9010460; doi:10.1038/s41419-022-04808-7)
Supplement: Supplementary file 2 — Supplemental Material [file 41419_2022_4808_MOESM2_ESM.pdf]

Uncropped western blots

Fig. 2A, Fig. 2B, Fig. 3A

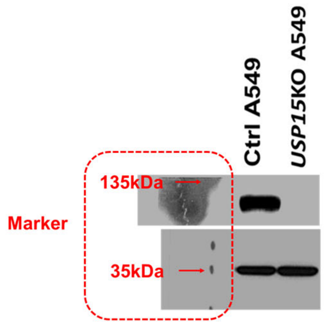

Figure 2A

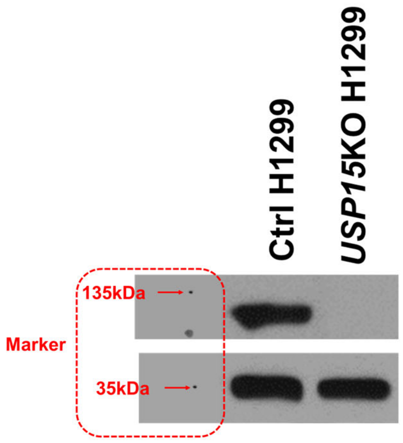

Figure 2B

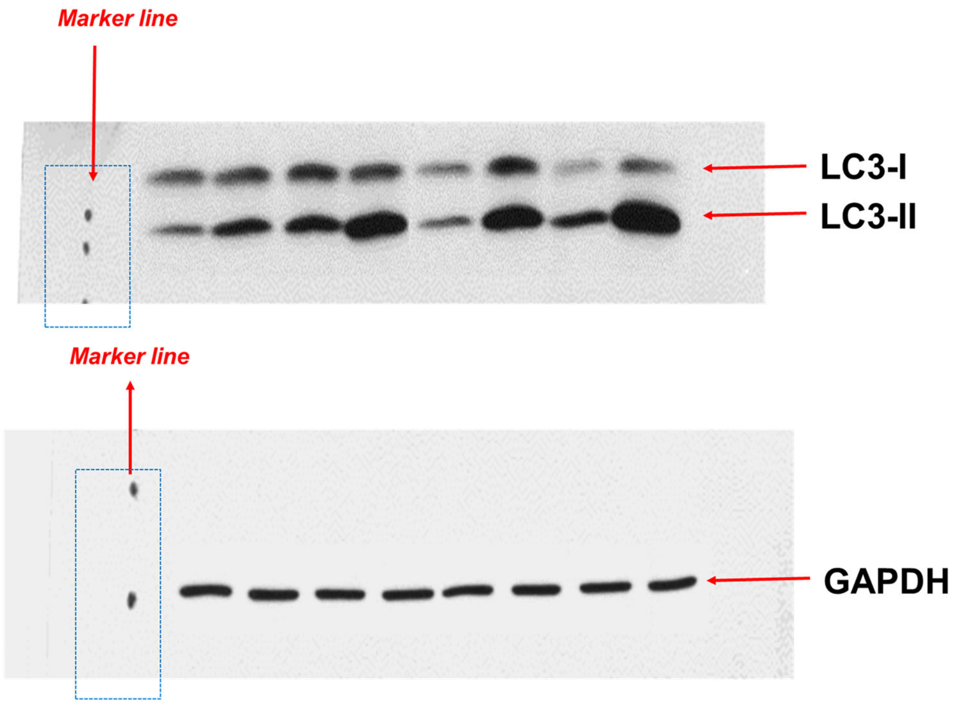

Figure 3A

Fig. 6A, Fig. 6B, Fig. 6D, Fig. 6E

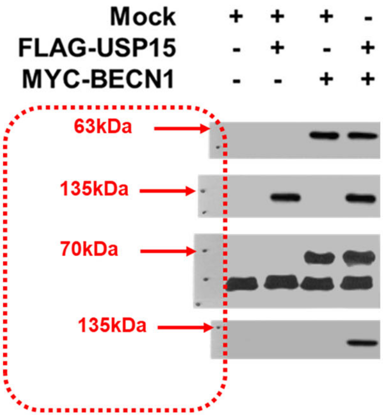

Figure 6A

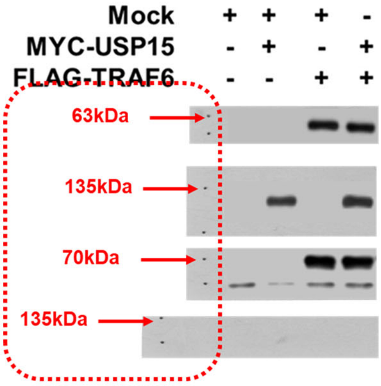

Figure 6B

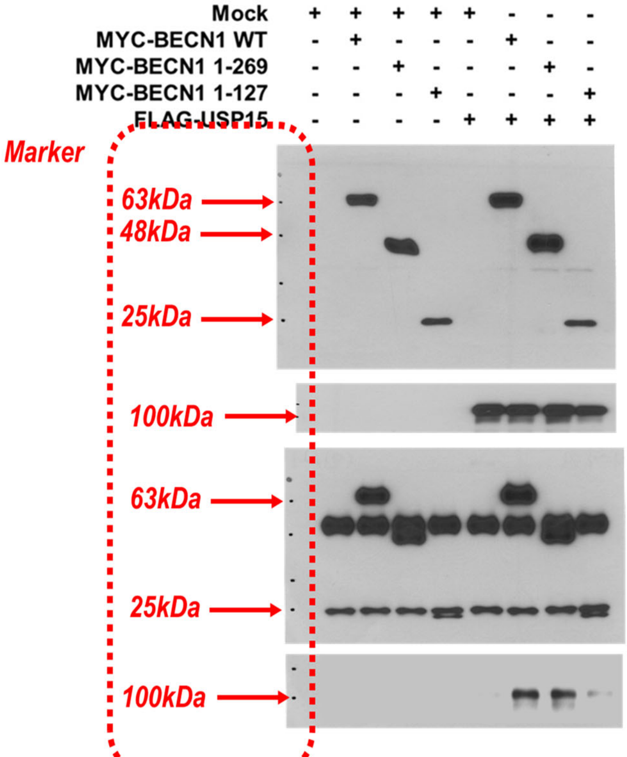

Figure 6D

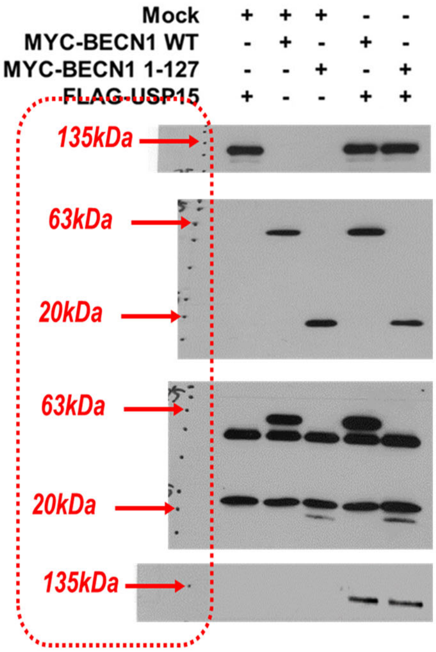

Figure 6E

Fig. 7A, Fig. 7B, Fig. 7C, Fig. 7D

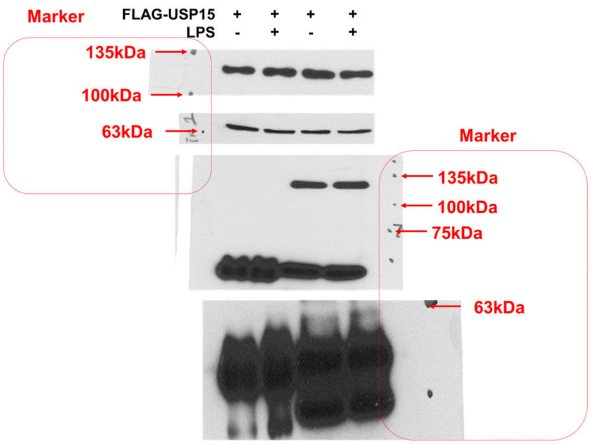

Figure 7A

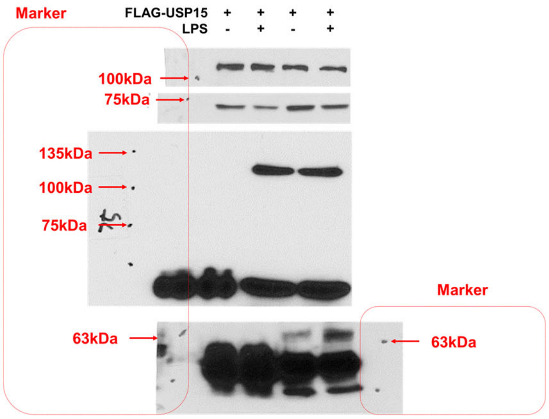

Figure 7B

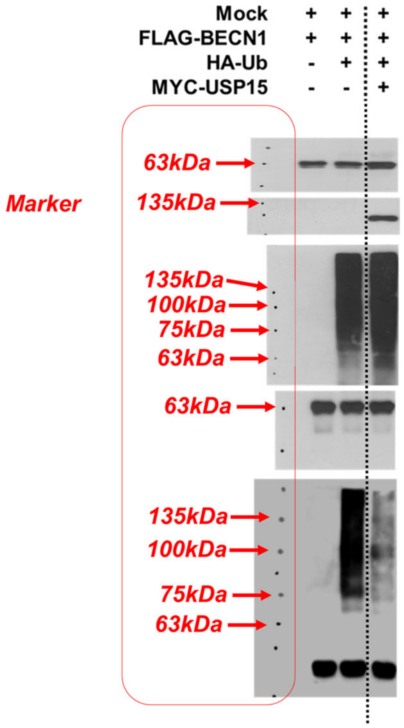

Figure 7C

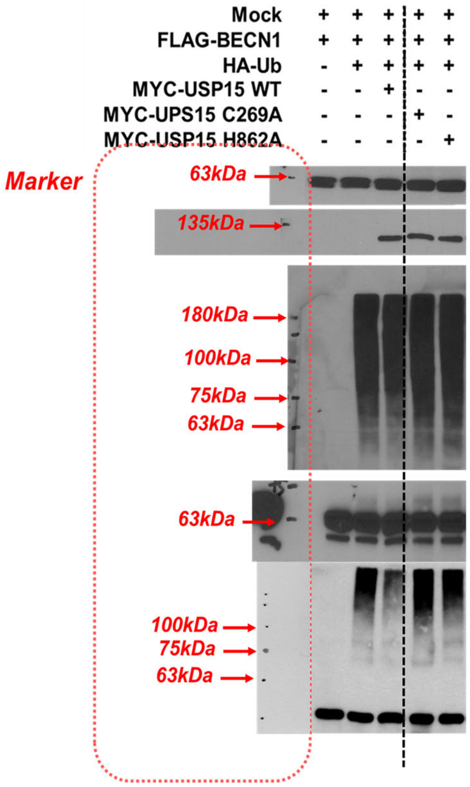

Figure 7D
