# Supplementary material for: USP15 negatively regulates lung cancer progression through the TRAF6-BECN1 signaling axis for autophagy induction
Source: Cell Death Dis. 2022 Apr 14;13(4):348. doi: 10.1038/s41419-022-04808-7 (PMC9010460; doi:10.1038/s41419-022-04808-7)
Supplement: Supplementary file 4 — Supplementary Table S1 [file 41419_2022_4808_MOESM4_ESM.pdf]

Supplementary Table 1. Up-regulated genes in LTT26 tumor patient are combined with those of LTT10, LTT12, and LTT35 tumor patients

TargetID (Gene)	LTT10 (Fold change, LTT10 vs. LNT10)	LTT12 (Fold change, LTT12 vs. LNT12)	LTT26 (Fold change, LTT26 vs. LNT26)	LTT35 (Fold change, LTT35 vs. LNT35)
LOC649270	-0.15199351	1.510176813	11.25898148	3.250808128
LOC644384	-0.47207656	-4.377117103	11.01936405	0.151790437
HS.278303	-0.885621147	-1.501731101	10.77512955	0.234288101
LOC729312	-0.526035271	0.235197062	9.736151893	1.27145229
RFX6	-0.987743203	-1.145486286	9.689885668	2.09368645
FSD1CL	-0.725091241	-4.160181881	9.666784777	0.067759232
C9ORF140	0.163469641	-1.00315218	9.206009655	1.163421649
LOC729581	-7.25598075	-2.520491858	8.878837261	-0.009665884
PHF19	0.869055314	3.734664011	8.707916533	0.908472345
LOC440456	-2.051194276	-0.131797181	8.6816857	1.112674831
LOC387924	-1.694478058	-5.196464636	8.596537748	-2.476857677
LOC100133404	-0.352820833	0.057600403	8.423315798	-0.052135999
LOC100134140	-1.883930584	-0.040077828	8.282366152	-0.591891816
MIR377	-0.775079786	-0.882473717	8.055131922	1.458773346
MFI2	-0.052218959	-0.772637324	8.003404015	5.861360591
HS.442098	0.128847568	-0.767316056	7.821176155	0.303922186
CRELD1	-2.916282245	-0.667821642	7.698249723	0.268825837
FAIM	-3.689485469	-0.347538065	7.641597834	-0.745779549
P2RY14	-6.91427615	-4.778145809	7.336105463	0.745647719
CCNE1	0.009361973	2.077865034	7.23083369	5.545177333
GOLGA8E	-0.58639021	0.735958663	7.225744839	0.628280455
HS.572268	0.602186292	-0.860482075	7.202607124	-0.494273354
LOC339742	0.723828258	-0.061757097	7.169086161	0.253329421
LOC100127891	-0.639274317	-1.192385991	7.143417383	1.626308294
NANOS1	-1.827927711	-2.423646996	7.12622015	3.569729242
MMP9	0.849144178	3.871799099	7.117217255	6.521763813
LOC643520	-0.776063547	0.642376399	7.064105466	1.853292271
CDC14A	-3.537683352	-0.170774239	7.041035803	1.293453133
DEPDC1B	0.25557073	1.324218451	6.97228104	1.719315259
ZNF483	-1.957348248	-2.581814406	6.894021503	0.782808587
DDX12	-2.054658902	2.169553767	6.893577362	1.864665395
HS.308073	-1.760073317	-3.637079525	6.805638238	-0.078469163
TRPV6	-0.003217508	3.429670233	6.727475839	5.548230203
C8ORF44	-2.601944301	-2.810543892	6.717418251	0.804126873
POFUT2	-0.487218456	-1.855879169	6.642469581	0.418465744
FANCB	-4.751359905	-1.073997049	6.54618632	2.271071042
FAM153A	1.181725721	-1.178865811	6.449185563	0.18414124
PRAMEF5	-1.394712707	0.194083533	6.423797465	-1.589110365
CAMK2B	0.276240894	-1.81706412	6.374037111	1.882585717
BIRC5	-0.462989581	2.166181556	6.332643843	3.19453639

SDHALP1	-0.951295849	-0.19313666	6.271987597	-0.785926556
CDC2	-0.418902456	0.924917779	6.269925788	0.810276383
RTBDN	-2.369602484	-1.28408014	6.22644001	-2.437749132
MIR92A2	-1.168010077	-0.223751774	6.217169512	0.394242525
SFN	0.727622078	3.442333083	6.164919562	1.833303577
HS.540335	-1.266986181	0.43344374	6.152546067	0.284002647
FOXK1	-0.57050203	-0.319872164	6.143539262	0.154344248
ZNF409	0.328255148	-1.73688012	6.121887557	-0.836215856
CNTNAP2	-4.505066996	-2.259634309	6.119549181	6.593107167
SLC4A8	-0.938946297	0.416118102	6.029801998	3.063662793
MIR506	-1.750688401	-1.244332638	5.996507033	1.178163471
MCTP1	-0.776075504	-0.725856739	5.929155594	0.903962929
LOC729454	-1.710698509	-1.834244647	5.898178089	3.123206853
LOC648639	-1.057981021	2.018107628	5.895986634	-0.22721359
LOC644366	0.607022204	-0.837997522	5.839809227	-1.663742979
CDC42	-2.616200443	0.027079956	5.83458806	-0.803596864
LOC653075	1.022089849	1.65200906	5.805986453	5.038160506
LOC100134073	-1.102688551	3.696298631	5.790458943	3.645141188
LOC646223	-0.318226061	-1.796410226	5.764901096	0.195085104
LGALS7	-0.74893458	0.29036508	5.726359846	3.687543663
LOC653174	0.501225111	0.525266992	5.715352047	-0.577858864
PACAP	0.198344919	-0.168934436	5.71051573	2.745091974
C9ORF140	2.21413771	2.169656556	5.6942844	-0.801421648
SNORD3C	-0.842038854	2.532939956	5.678166421	3.665165978
BTBD17	1.713408055	-1.137503287	5.640857704	3.455437232
HES6	-1.27308881	-0.731626432	5.624936107	1.098555188
SYT13	4.486514346	-0.794418798	5.604646469	1.148812328
KLK8	0.267510319	3.021849434	5.565926428	-0.376762604
LOC728178	-0.866518981	11.9260063	5.550781498	3.548480434
ENPP7	-0.265135926	-0.081119099	5.537464001	-2.8032324
SERPIND1	-4.679720495	2.232601113	5.520907578	0.312414011
CELSR3	-0.589045109	1.723019994	5.500998699	2.888895641
ZNF268	-0.69944085	-2.864661748	5.489542095	-1.467162698
FLJ40113	0.815361314	0.969394289	5.471075539	-0.019114734
LOC442480	0.721545488	-2.299028248	5.407421409	1.467783998
C6ORF27	-1.126699213	-1.840315347	5.399743495	-0.086452692
HS.47453	-1.170874158	-1.162385444	5.398535444	-0.701593393
RAD54L	-0.906537715	1.925035098	5.397047037	6.218537023
GTF2I	-0.989888213	-0.0474715	5.386045553	-0.047648765
ZNF497	0.953091534	1.772928868	5.320023373	5.942481485
LOC728910	0.117158612	-1.141927281	5.31700275	3.557335722
TTK	-0.175087442	1.361670449	5.282002689	1.977816515
USP32	-1.080771139	-0.125668647	5.277587222	-0.290348798
LOC650114	-2.625920147	-3.039260003	5.268262026	0.010832815

SNORD3A	-0.722221392	2.524994595	5.230300889	3.285885397
CA9	6.055343282	5.940020128	5.222037711	2.666099745
SCG5	-0.669223232	-0.183150546	5.19874653	3.058792923
DMD	-2.783875675	-0.174314312	5.197004057	2.145445211
ARPP-21	-0.893987892	2.043118126	5.180498489	-3.352732393
C1ORF170	-1.58305827	0.020249254	5.152796243	-0.37125399
LOC441126	-1.492232761	-0.946959892	5.143414282	0.398646206
DDX11	-1.065702891	0.32885802	5.119208634	0.560765866
SNORD3D	-1.357781946	1.67362247	5.026604463	3.309753637
ESPL1	0.744808552	1.485312089	5.009506839	1.06444392
LOC100130508	-1.898762573	0.075028947	4.975810567	0.264173702
PODXL2	0.487867911	2.48323315	4.966751986	4.047070101
LOC440895	-5.410794978	-1.541330179	4.959999276	0.23246776
LOC100132564	-1.442558578	-0.119890352	4.949516088	4.992950575
NOXO1	-1.706936606	0.929987137	4.940526339	-1.749310641
CALML5	-1.155784425	-1.103138053	4.921178405	8.80177805
LOC649667	0.580449138	2.386018164	4.888659156	-1.738930581
CREG2	-0.989234268	-1.402212393	4.88657859	-4.027963933
DDX12	-0.519095704	2.105243291	4.877547656	1.740832646
LOC100130312	0.46189497	-1.004222663	4.874512389	2.32126669
LLGL2	-0.309542112	0.977789752	4.847588617	3.334791691
SNORD56	-0.970062764	0.525906521	4.840404776	3.193571181
FGL1	-0.921689981	0.135841396	4.797340145	-1.142192812
PLEKHH1	0.873159047	-1.493980149	4.796684248	4.355583095
LOC644092	3.24922605	2.637609383	4.79589707	1.01518425
LYPD1	-1.776711497	0.844300303	4.793117987	0.87287648
LOC653555	1.313875782	-1.586452772	4.78048928	1.667867864
PROM1	1.922263729	-1.099993942	4.7716873	1.455034431
LOC730087	-1.146811933	2.944269361	4.767972028	-1.29863917
MYO1A	1.41328164	-1.722016347	4.758691346	-0.405857886
PRDX2	1.831270822	-0.298472184	4.73530131	0.624847304
LOC100132101	-1.009772484	-1.558971347	4.70579579	1.980171924
LOC653468	-2.780038499	-2.25249863	4.702686715	2.498392445
FLJ35816	0.108139691	-0.955225954	4.692057359	-0.175366492
TAF4B	-1.047731572	-0.858740405	4.690571003	0.133261483
CENPF	-0.064636369	0.498914944	4.683105393	3.111672068
CIDEA	-4.367457861	-1.137772904	4.664936495	0.053985318
LOC728755	1.217864417	-0.807766381	4.65804256	-0.995729843
NAP1L6	-2.047452792	-3.829269028	4.642588558	-4.204847539
LOC650076	0.319433335	0.32519383	4.634825187	0.770513105
MESP1	-2.340590947	3.647646804	4.61410956	3.431095456
PRR15	0.536136582	-1.60814076	4.612688645	-0.654308321
HS.566857	-2.016184534	-1.160199042	4.606530114	-2.109805111
WDHD1	0.6558624	-3.389358791	4.60635934	3.977573024

LOC642965	0.287516711	-1.428670942	4.596143684	-1.358566364
LOC100134400	-0.617155145	-0.333877078	4.589962593	-0.057464525
TMEM80	-2.689820539	2.86216161	4.589411944	1.839410785
FGA	0.39270237	-3.512057746	4.581592284	-0.998063149
LOC148766	-0.489594078	1.177218603	4.579481673	1.072717102
CRYBA1	-4.221933534	3.049860744	4.563027282	-0.10678561
UBE2C	0.212759385	1.223136628	4.548709059	2.99881419
C9ORF100	2.499680033	1.459297333	4.512210804	2.492911397
LOC440456	-0.514181675	-1.174901499	4.478603348	1.612399353
KIF18A	2.155260776	3.55890279	4.474473721	0.699943241
SMO	-1.558468214	-3.358939557	4.470811	-0.180900343
LOC647281	0.823599652	0.807542187	4.470787518	0.24999715
ATPBD4	0.317063238	-0.714457589	4.464606134	-0.284775563
ISL1	-1.387909321	-1.920560365	4.454661077	2.24422709
HELLS	-0.615179857	-0.138043783	4.444164935	0.261814082
LOC390594	-0.677628933	0.661515207	4.431732692	-1.618789873
LOC653471	0.273892292	-0.572445207	4.427830784	0.596077065
CSNK1A1P	-0.650388649	1.614443516	4.426091287	-1.977086035
TMEM145	-2.134137057	-0.827205082	4.421603653	1.524830198
LOC647042	1.26920763	2.9715043	4.405911939	2.424005385
PRR22	0.020862054	-0.565107412	4.388232082	1.745289789
LOC100132113	-0.598513073	-1.812095387	4.385213044	4.18684772
SPATA19	1.583996956	-0.678127218	4.38035655	-0.98850018
LOC145837	-0.29668923	1.773242595	4.367872488	-0.602129198
MIR208B	-0.364056623	1.088678488	4.366234044	1.279784313
FBXO24	-0.57671623	-1.47462691	4.358749195	-0.121368913
LOC646663	-2.767548758	-0.1200144	4.351682588	0.43572308
ADAM8	1.753071198	2.573674428	4.349781449	2.6139363
UBE2C	0.483422298	1.000710382	4.345633664	3.038892154
HS.543983	-1.680281927	-1.621416505	4.344062292	-1.026005019
LOC652554	-0.425171659	1.537210849	4.340538753	-0.772489464
LOC283683	-1.888491359	-1.014470724	4.334708462	-0.273378527
CLEC3A	1.211498038	-0.065437736	4.334404772	0.455724127
VCX2	0.065151726	-1.22781665	4.328810027	0.784355756
RGS17	0.248861948	0.821949909	4.315526557	4.266917425
DNAJC12	-0.174102881	-1.462475712	4.307729925	0.472516035
FLJ22184	-0.140381285	0.489536281	4.297744252	1.651335911
GABPAP	0.740105069	-1.957560642	4.297223016	0.371395769
PRR7	-1.6430315	0.962625839	4.275127367	-0.10629399
HS.555255	-1.40942819	-2.510317005	4.272456767	-0.225587231
KRTAP23-1	-0.279208432	-0.886363517	4.261897143	-0.212708882
LOC728509	-0.527979424	3.765590976	4.257657194	2.855265076
PLUNC	6.592115495	-2.692569822	4.255201507	5.915631739
AAA1	-0.494383524	-0.197796307	4.250800448	-0.594781622

PRR15	-1.060779112	-1.820508465	4.247829726	-0.469009413
SLC29A4	-2.79438483	1.129884762	4.242568233	0.134268084
LOC100132292	-1.35987185	-2.650023901	4.238575961	0.858226086
HPX	-0.889247932	0.302735267	4.238036099	0.055118696
LOC727735	1.253831356	-0.758038565	4.237015429	1.900983891
HHAT	-1.557427645	1.071251495	4.23144327	0.94769861
LGI2	-2.376780797	-3.052617284	4.228666098	4.787028033
CCR2	1.603137028	0.19364203	4.224408218	2.664219888
RCVRN	-3.549263126	-0.951479271	4.21425935	-0.563937788
PTH2	0.872270314	-2.839766825	4.202411845	0.221337875
LOC654208	-1.157648695	2.242738537	4.200656812	-0.019750097
LOC100128615	-0.23946007	0.293495766	4.188528712	2.093081488
LOC100134360	-2.894642127	0.187075878	4.187020067	-0.085653367
LOC651017	0.102516879	0.15861265	4.186847927	1.310478084
LOC646993	-0.93360775	0.644888499	4.145297267	1.300982673
LOC654342	-1.059021987	0.139213022	4.137656934	0.736304563
MMP21	2.238631592	-1.42274994	4.136990073	0.618713886
MARVELD3	-1.577985444	1.408186757	4.133172798	2.119512541
SSX6	0.236908592	-2.396646768	4.130782094	-0.230504102
CCNB2	-0.566500863	1.414249367	4.116155194	2.680420877
TFAP2A	1.642522354	0.907654043	4.108089018	4.272968272
GRAP	-1.264788077	-0.815330996	4.107278862	-0.627278075
ASPM	-0.475452905	0.012865401	4.107121954	2.923884711
CTAG1A	-0.541611009	-2.551190503	4.105285865	1.643549212
HYDIN	-0.651933099	-4.569188419	4.100504312	2.172757217
IL4R	-3.809491015	-8.10613982	4.092763707	0.306608781
HS.26579	-3.014175491	-4.893385252	4.088026648	-0.205869647
PCNXL2	-2.041785641	1.90192502	4.08168623	1.797708782
LOC441136	-1.241081538	0.040516047	4.062756989	0.505954782
LOC649346	-2.263648186	-0.647370815	4.042268595	0.454212845
C20ORF103	-1.10920805	0.782496266	4.038528404	2.646166723
SNORD99	0.065778529	0.905869517	4.030152766	2.81033575
LOC642393	-1.681574601	1.849578995	4.029515046	0.759331275
LOC390933	-0.632436434	-2.259609555	4.010049152	0.148666676
HS.436189	-1.360408116	5.542231178	3.992718497	0.099333359
SMOX	6.494630562	-1.262821135	3.988987551	1.78631358
RUFY2	-0.263809445	-2.136763015	3.987182657	-0.134720752
NOS1AP	-0.527884152	4.247739401	3.970330954	2.676434381
SPAG1	-1.534861326	-1.372387191	3.95344434	-1.179287399
GNG4	-0.35112177	0.834768887	3.951314913	3.958217133
LOC100133191	-0.411520103	-0.847938257	3.942602744	0.850689313
C20ORF194	1.991353566	-0.327134315	3.923892759	0.875867221
LOC100134587	-0.697370209	1.49332358	3.917255603	2.239008053
DTNB	0.379050769	1.447808927	3.908542855	2.542793933

NARG2	-3.721509605	-2.897004273	3.906359429	0.200095402
OR6N1	-1.275400796	0.852239693	3.901529006	0.823407874
AURKA	0.284228069	1.461907646	3.894845483	2.086459381
FLJ45513	-0.629937049	-0.206941226	3.892251783	-0.132539052
LOC100133077	-3.110741067	1.108714448	3.890935713	-1.018664204
NUP98	-2.995200528	-1.522179249	3.8824927	-2.556884452
DNMT3B	-2.095680934	0.437693776	3.873907795	0.751508259
SNORD57	-0.982201955	-0.34445602	3.872825745	0.718681054
HS.145444	-0.775266031	0.277478689	3.869792067	1.36588615
THEM5	0.165729432	1.967258078	3.853556595	-3.273288934
GALNT6	1.372630526	0.89904898	3.84639209	2.324661073
HS.568058	0.689465944	1.495187021	3.844220869	0.597556471
POLR3D	-2.490096607	-1.305378685	3.842221042	0.00124635
C15ORF54	0.485627403	0.183243477	3.834018276	0.568333849
KIF15	-0.285750901	0.430209629	3.833550019	2.233968851
LOC652369	-2.117423518	-0.052960099	3.833106782	-0.552759252
LOC649660	2.81632644	-0.02076799	3.828734452	0.104454614
CLK2P	1.612717664	0.059799617	3.827565004	-0.384736867
LYG1	-0.69680281	0.345902494	3.822944008	2.184735562
LOC653610	1.572795226	-0.053446256	3.820001966	2.594146417
LOC648628	-1.885352104	1.182846271	3.819870012	-1.765271202
FAM136B	0.11860202	-0.658674693	3.818692621	0.844573995
LOC100130673	-1.894686969	0.122577293	3.804201663	-2.268780584
HS.544145	0.658324601	-0.516957189	3.802150807	1.910555801
EZH2	-1.358868282	-0.132364206	3.786390617	3.51901821
KIFC1	-0.21290357	1.344459512	3.782920543	2.919418652
COL7A1	-1.117338467	0.87045124	3.782152081	2.760971879
LOC729296	0.168638523	-0.92090481	3.774283902	0.141430255
LOC729668	-3.27136832	-2.057096865	3.771778313	1.129842126
NBR2	-1.228700366	-0.85936805	3.755241421	2.459412337
RALGPS2	-0.898398916	1.065722104	3.754253132	1.319405231
LOC646970	-0.420998096	-0.954074103	3.750283788	-0.043827555
LOC730546	-0.595899339	0.994120033	3.749196924	-0.064650531
CLECL1	-0.44776145	-0.46077368	3.74841707	0.812368947
LOC400986	-0.857806243	-0.952072976	3.745171196	1.410585313
LEMD1	0.250155604	0.023662971	3.740550829	2.056323864
UGT1A9	-0.111788137	1.851123979	3.731618642	-2.698325704
HAGHL	2.059575187	0.766440459	3.730456756	1.477753232
PROM2	0.784030351	1.613252639	3.723995096	3.111637287
ZNF114	-1.559853734	-0.385661464	3.703665712	0.746310497
HS.576042	-1.321701734	-2.472929788	3.702164701	-0.10126285
LOC728648	-2.730615191	1.32415808	3.691584	1.353096575
USP45	-3.213775463	-0.549380452	3.682929925	-0.096881184
SCN9A	-1.397396554	-1.283838126	3.682880218	-2.019345706

LOC642397	0.609829295	-0.282579479	3.681583324	1.116583971
WFDC2	5.432675172	4.112449373	3.670251411	3.610661907
RCOR2	-1.260635648	-2.148396905	3.663442744	2.157412928
MIR26A2	-0.973172652	-1.912369867	3.662995746	-0.436930372
RTKN	-4.318900053	-1.547430687	3.659532683	-0.862198885
LOC645682	-2.638385647	-4.999566926	3.65950609	-2.573270987
LOC644466	-2.806671317	-1.747240182	3.658952049	-1.398851946
EYA2	0.036313415	-1.941713517	3.650709577	1.918698842
LOC729389	-1.609490101	0.292818961	3.646867027	1.620556292
DPF1	-2.110584848	-2.436356456	3.635505675	0.173626844
PHLDA2	5.598503102	2.732579125	3.628689527	1.828437788
CCNF	-0.857422022	0.140348786	3.624056383	1.390406853
LOC641958	-2.495677788	0.256111589	3.621424151	10.04102639
LINCR	-1.192452981	2.755895734	3.619070946	2.044647559
C14ORF181	-3.462836008	0.84590254	3.611021942	1.532459168
PMS1	-2.300164968	-2.512319004	3.605147805	-2.546207556
ERN2	4.780481886	3.890220041	3.602654082	4.240995762
DPRXP4	0.340805065	-0.552448049	3.584025673	1.698571167
LOC653576	0.080470365	-1.450554669	3.583307922	7.651808379
FAM83A	3.199090533	3.186924007	3.580316098	8.350634551
OSBPL7	0.717475116	1.599705561	3.576852811	1.561462067
ETV4	1.883346557	2.628710681	3.566510957	2.968531407
PCDHA6	-0.091667538	-0.827517874	3.560589535	-0.287079747
ANO9	0.56542666	2.542136094	3.557730571	1.781282611
NFAM1	-2.623687041	-2.13946966	3.534428893	0.77988006
TOP2A	-0.227593768	2.112843763	3.532901932	4.569881563
HNRNPCL1	0.830137471	-0.863633133	3.51576658	-0.75364108
MYO7A	0.815599335	3.164744785	3.5088399	3.505698815
NNAT	-2.140464135	-3.526376268	3.50321085	-0.105473063
FBLN1	-2.795267754	-2.456358335	3.498582172	-0.102723522
CTLA4	-2.071359643	2.260908335	3.493659558	3.203449825
BRI3BP	-0.224545322	0.035824308	3.48714341	0.864028878
KIF11	0.318614735	-0.646746259	3.480263595	1.611454793
CDC20	-0.023610084	1.900325522	3.476446495	2.738798853
LOC649841	-0.342175713	-0.569997951	3.470713192	1.925968694
LOC100128354	-3.07824393	-4.459327718	3.4683617	-0.248886853
CDH20	-1.600760591	-1.943020819	3.466193784	0.063213493
LOC646762	-0.034541626	-1.501772372	3.463355227	0.453559507
LOC728308	-1.327126276	-1.464620009	3.455612604	0.400333759
MGC48637	-0.089687615	0.136994679	3.453050438	-0.768241813
LOC391039	-1.56209937	-1.673039109	3.44267223	-1.914419496
HS.319406	-1.706712967	-1.931229818	3.439578328	-0.150337495
ECM1	-2.305683044	-0.215994078	3.434226582	-0.208660396
BAT1	1.565751408	2.132529532	3.42938276	-1.080768672

ASB7	-1.463617704	-3.472972517	3.428016137	0.517606757
ARHGAP22	-1.262191453	-1.427547671	3.423172782	0.672045
MCM3APAS	-4.044423	-1.563288292	3.421465153	-0.870048765
LOC150759	-1.169014875	-1.098798509	3.413194621	-0.03996243
BUB1	-2.00737107	3.442347354	3.410487316	2.318846801
CPT1B	0.3138512	1.888847925	3.410144464	1.36574641
OR6B1	-0.450086056	-1.151886767	3.409115831	0.716191236
MCM7	-0.272250985	-2.610673348	3.407050078	2.51193109
LOC202134	-0.692731387	0.765069178	3.406569366	2.387812655
TMEM45B	-1.542090591	-0.342679357	3.39970648	1.240525775
MMP13	-2.664702081	0.248298561	3.399446098	1.502746741
LOC100132911	0.222586267	-1.948694109	3.371570813	-4.690460073
LOC389816	-1.065746918	1.0670814	3.367802277	2.177753769
TROAP	-1.006288785	1.001506831	3.366499071	2.11058639
BCOR	-3.314204963	-0.712089079	3.366467031	-0.160549828
LOC731999	0.054099002	2.437897412	3.364479691	1.859603836
B3GN-T6	2.760914468	-1.470468721	3.361827689	0.610741299
LOC642587	0.709623734	-0.787918412	3.357186157	2.318108911
SPC24	-0.807125435	0.803802907	3.356705566	1.446928353
LOC100132942	-0.751740942	1.94717397	3.354722445	-0.756300968
LOC727833	-2.422905491	-0.347209767	3.353717936	-0.670561921
LOC400013	-2.753587008	-2.630641456	3.349041038	-0.050192063
ANKRD36	-1.060126455	-2.576341599	3.343347827	1.800092109
SLC39A13	-3.830863141	-3.373739956	3.341845105	-1.338300129
LOXL1	-1.498190395	-2.463465178	3.34094866	0.413893943
LOC729956	-0.758812908	-0.102434875	3.340374617	-1.076640007
PYY2	2.171689228	-0.508742807	3.338987761	-0.108838777
LOC729858	-1.244467586	-0.913428847	3.328525808	1.66665297
THYN1	-0.918526167	-0.95293687	3.327729986	-2.825031823
RECQL4	-0.392790694	1.016875725	3.321980866	1.71862801
LOC646278	-1.498788039	-1.492465231	3.320924674	0.15761235
SFRS14	-0.082352973	0.416818615	3.31966575	0.954743822
PLG	-0.433440841	-0.105625494	3.318941947	1.591722342
SNORD125	0.363088399	-0.597823254	3.312209113	-1.008262161
LOC402509	-1.776808574	0.860336544	3.31188427	0.168314327
SCARNA3	-2.574820076	0.791608101	3.311155687	0.513801876
HIST1H2AJ	-1.78648549	-0.97399661	3.290061794	-0.224684422
CSMD2	-1.072103611	-2.026990596	3.288729285	0.635031635
CAPN12	-1.609637843	0.756851154	3.270842868	0.474724971
TTC9	-2.119191073	-1.15039864	3.266530381	-2.372889534
C12ORF48	-1.647244477	-1.582848619	3.253474781	3.241014673
FUT10	2.176637315	-1.048610192	3.247270429	0.572977758
LOC729954	-0.989715414	-1.073917678	3.245940974	2.249226274
LRRC26	-1.456083906	0.046307194	3.242151287	2.059535453

CCDC7	-3.362648776	0.695757273	3.239678818	-0.099339513
HS.389313	0.291030355	-0.97263113	3.235697253	1.408827846
LOC642367	-0.921162355	0.523581172	3.211551363	-0.060707431
COL1A1	-1.114497092	0.098962867	3.203331186	4.657687937
LOC100128386	-1.40637503	-0.383691613	3.201474096	-0.835511143
FLJ40113	-1.348978054	0.671442452	3.193745897	1.09394978
WDR87	-1.08876962	-2.458762957	3.19054166	0.781261853
CYTH2	0.417389249	-1.106122857	3.188728047	1.360242338
HS.473191	-0.852208465	0.691662287	3.187166247	1.183374187
FGFBP1	1.194056751	-2.039519013	3.180553971	3.565959238
GOLGA8A	-1.117304178	0.682314901	3.180211744	0.993684239
MYO19	-0.793182769	0.711181103	3.177221187	2.294276607
UPK1B	-0.975472312	-0.91767502	3.177093655	-0.718255329
LOC100129213	-1.770759177	1.716870773	3.174911447	1.198636078
RNFT2	-1.518262897	-2.173872149	3.174692184	1.167317007
MMP11	1.953418307	2.085707617	3.165695156	5.07243987
LOC91431	-1.200692685	-0.371356812	3.162904859	-1.111622345
AUP1	1.625127669	-1.467476804	3.161480321	0.600367901
PON1	-0.97837625	-0.292889618	3.160184319	4.524710201
TRPM8	1.873092606	-0.667537074	3.151325707	1.331221561
PGK2	-2.955841445	0.292025619	3.150835274	0.581238182
SLC30A4	-1.406036281	0.128188107	3.149738178	0.495525351
LOC645330	-1.465208711	-0.774180648	3.147926947	-0.296388173
NXF4	-1.011677007	1.724706231	3.143203423	0.346530177
AVL9	-1.102253166	-0.463189404	3.140601054	1.972961818
LOC401321	-0.918011084	-0.913864905	3.140421029	-0.268090608
LOC100132159	0.110036266	-1.416045704	3.138480126	-0.187242186
MIR1180	0.418516459	-0.145253786	3.13828209	0.8727556
LOC100129186	-1.548259392	2.479172831	3.136362908	2.721108387
EPHB3	-0.784643383	1.073413119	3.130254591	1.880881355
CHEK1	-1.642316008	1.042725187	3.130128005	4.316705034
HS.544507	0.393045311	0.15008978	3.126335932	2.772860903
TFAP2C	-2.896964131	-0.08834805	3.123215054	1.66757978
TAL2	-0.622365844	-1.306133901	3.120265753	-2.43681425
FLJ20674	-1.798226991	-1.569123314	3.119000629	1.592926754
LOC649200	1.234342063	-0.467645414	3.113632944	2.732961537
PROSAPIP1	-0.932914861	-1.037103668	3.111211987	1.060099076
HS.252668	-1.04195885	-1.001668302	3.106488023	-2.372267969
TMPRSS5	1.014576865	-2.820812472	3.098583086	0.851882594
MYSM1	-3.690530224	-2.401616024	3.098497111	1.848353236
LOC100130116	5.637355276	-1.965740973	3.097822598	0.735450786
BCL7C	-4.168250492	-0.322772023	3.088826351	-0.306200179
MGC22014	0.391603309	0.68243108	3.088171978	1.268345509
HS.439363	-2.19233477	-1.06912615	3.086328663	0.211327689

INE1	-1.809383918	-2.408806184	3.085310573	-4.609142114
SERINC2	0.361045025	1.027929147	3.079592099	0.818528316
SLC5A8	-0.581386837	0.001672317	3.079020464	0.991465755
UBE2QP2	-1.781227495	1.111353429	3.078117356	-0.787762229
LOC339692	2.220364745	-1.871006347	3.075838111	-0.089012424
HS.564510	-0.983974211	-0.215997166	3.07064039	0.097436437
NCAPH2	-0.253539741	-0.656462837	3.070108097	-3.08452789
LOC400553	-0.100101806	1.044932186	3.069411288	-1.220222965
DIO2	0.075623763	-0.980016727	3.065293884	2.883392499
CDC25B	-2.604064825	0.02743503	3.064249777	-1.968914301
WDR86	-1.927499557	-1.211349813	3.063761503	1.688940217
LOC100133263	-3.142004976	-3.271623053	3.063030974	1.24609218
LOC650498	-2.04756779	-2.699550793	3.058992321	0.87751241
C20ORF94	-1.387214126	0.082437951	3.056981511	1.485903027
CASP8	-4.46084296	-4.83462755	3.055750984	-3.442465387
LOC392583	-1.912470508	-1.041612836	3.044721875	0.43757886
MRVI1	-0.785812045	-2.061028051	3.043331736	-0.265889706
LOC100129064	-3.333525236	-1.550250759	3.025265558	1.889814408
LOC124220	1.456052634	-0.55284598	3.022181288	0.471231609
KIF2C	-1.067482975	2.819642915	3.016830224	3.373908376
GSDMB	2.014116008	0.755915491	3.016526482	2.712147108
LOC645367	-1.516007648	2.210216068	3.014861718	1.155429932
HS.563922	-3.443100344	-1.247093002	3.013896967	-2.152122443
NFX1	-2.345118829	-0.707022993	3.012330315	1.476721606
LOC440345	-0.562206378	1.308342827	3.011404519	1.227525723
LOC85389	-2.811791353	-0.216032513	3.00777317	1.509323605
SPAG5	0.211311217	3.454111723	3.003678748	0.710802075
