# Supplementary material for: USP15 negatively regulates lung cancer progression through the TRAF6-BECN1 signaling axis for autophagy induction
Source: Cell Death Dis. 2022 Apr 14;13(4):348. doi: 10.1038/s41419-022-04808-7 (PMC9010460; doi:10.1038/s41419-022-04808-7)
Supplement: Supplementary file 5 — Supplementary Table S2 [file 41419_2022_4808_MOESM5_ESM.pdf]

Supplementary Table 2. Down-regulated genes in LTT26 tumor patient are combined with those of LTT10, LTT12, and LTT35 tumor patients

TargetID (Gene)	LTT10 (Fold change, LTT10 vs. LNT10)	LTT12 (Fold change, LTT12 vs. LNT12)	LTT26 (Fold change, LTT26 vs. LNT26)	LTT35 (Fold change, LTT35 vs. LNT35)
FCN3	-3.32114575	-6.650156822	-15.53550572	-4.938997031
TIRAP	-0.656848652	-0.809243876	-11.59299129	0.343193889
CTSG	-1.981226422	-2.600648527	-11.43677293	-2.054269763
GIMAP1	-2.703045278	-6.626820898	-11.36877816	-1.005232415
SVEP1	-3.1038928	-7.354731598	-11.30247091	-2.486790356
CLDN18	-0.627412749	-6.81328365	-11.2356595	-4.057344116
LOC644734	-2.011924472	3.225877483	-11.22157927	-4.740467079
ZNF571	-2.122718896	-2.940462708	-11.22083092	-1.992273632
ALDH1A1	-0.238449321	-1.632470262	-11.15339745	-2.442869173
ABLIM1	-2.080764384	-4.376606988	-10.9539386	-2.455079325
RAB5A	-1.195896682	-3.181112009	-10.94869652	-1.9696933
FCER1G	-0.322530818	-2.480640633	-10.5723862	-1.601783043
B3GNT6	-2.317891229	-2.032911728	-10.39526724	-2.681541335
C14ORF128	-2.967259831	-2.082282787	-10.39126281	-1.337647891
WTAP	-2.869727405	-3.408994844	-10.32681689	-1.605949862
COLEC12	-2.408434107	-5.606262503	-10.24338499	-2.88383852
DMRT2	-3.497488786	-4.756863883	-10.24251335	-1.647417419
TMEM1	-2.405997533	-4.817179162	-10.21196825	-0.481141291
C10ORF32	-2.474018592	-4.384796087	-10.13825152	-1.882193906
SQLE	-1.403697729	-2.573898218	-10.11155624	-4.1665797
HIF1A	-2.273330583	-2.691124215	-10.09441834	-0.797659192
EPN2	-0.273430684	-1.566304804	-9.979007639	1.806057811
KLK5	-0.581670408	-7.469152493	-9.961605146	0.304702422
H2AFV	-3.328356786	-3.441078745	-9.914080112	-2.05484865
C4BPA	-1.345419395	-5.565714179	-9.765806775	-2.143327372
HS.545536	-1.616898252	-0.896992955	-9.740049168	-0.596154137
FABP4	-5.618904037	-11.2048116	-9.672257756	-6.823644396
PDZRN4	-0.113627345	0.532134161	-9.617798164	-0.202948545
SCGB1A1	-0.703907486	-4.325165709	-9.600357233	-8.02938584
MGAT4B	-2.901160446	-4.195902792	-9.525420954	-1.600909329
SIRT2	-2.166255899	-4.033727219	-9.517229102	-1.78015167
ITGA6	0.757607086	-1.529256195	-9.509151574	-2.888050892
TNNC1	-2.430875713	-5.100787719	-9.496628208	-4.415263408
LOC729217	-1.832246769	-3.374817037	-9.458276934	-1.651044603
C11ORF1	-1.372473807	-1.306389836	-9.427866277	-1.639938688
SRPX	-2.931199638	-7.694840395	-9.335327926	-2.680635158
HS.558072	-2.038632161	-5.453653095	-9.321936719	0.022991628
GPRC5A	-1.889323957	-6.339202492	-9.296498678	-3.060372037
PPP2R1B	-2.044287899	-1.745531888	-9.228182515	-0.52629526

IL17D	-3.660383424	-7.496588723	-9.169210225	-2.958474772
HS.164221	-1.837330584	-3.380645915	-9.114342216	-1.537527603
EFEMP1	-2.333707024	-5.342822722	-9.096071157	-3.321683002
LOC401317	-1.364886865	-1.576607886	-9.020412501	-0.463768223
FLJ32063	-0.090825156	-0.028959135	-8.991892073	2.798365511
CRTAC1	-3.325378971	-7.596887423	-8.989533198	-3.779360897
EEF1B2	-1.26946438	-3.74228897	-8.987321936	-2.37560643
LOC402057	-1.759306128	-3.992582459	-8.959082812	-1.570748749
CLDN18	-1.576801793	-7.79102021	-8.954657688	-5.439697626
PLA2G1B	-2.914329133	-3.651687688	-8.876110782	-6.022191207
FOLR1	-2.375251401	-0.912275387	-8.854634616	-2.704544637
PDPK1	-2.43921565	-4.393129625	-8.811101531	-1.481113023
CCL13	-0.943899928	0.298429421	-8.796470829	-0.157357811
METRNL	-1.075743259	-2.646748245	-8.772129382	-1.782101312
EAF1	-2.64116223	-3.364239851	-8.761811749	-0.958427201
CUGBP2	-2.005389821	-2.761303708	-8.75854479	-0.853334977
LOC729920	-3.207073604	-3.170792453	-8.750931802	-1.883757937
LOC441996	-1.447716194	-0.259346499	-8.697749762	-0.140558818
OSGIN2	-2.120755782	-5.184013604	-8.696313542	-4.133181297
LIFR	-3.135802625	-3.963865305	-8.666640735	-5.509990589
DHX29	-1.74352451	-1.744275561	-8.64481609	-1.118574721
CD48	-1.141724616	-2.651987139	-8.634584428	1.052102027
CAV2	-3.361349495	-7.061679642	-8.597376793	-4.152386133
CD9	-1.544317531	-4.330023742	-8.586532342	-2.39983598
LOC649143	-0.938804982	-1.7194645	-8.462155993	-1.523390792
CLIC5	-3.581121354	-7.01904378	-8.436524813	-4.234411245
HS.541057	-0.703329051	-2.471774848	-8.426546213	-1.009384508
ZBTB11	-2.605190233	-3.90906098	-8.416468234	-0.79617062
LOC391475	-0.989358977	-1.305575562	-8.350517092	0.158491273
LOC647081	-1.344417801	-3.489344774	-8.339295004	-1.214324336
ABI3BP	-3.223885434	-6.516260177	-8.312197874	-3.357294219
CD37	-0.749122331	-2.439426375	-8.277461762	-0.184132476
SEPP1	-2.126573692	-3.648460853	-8.25401476	-3.305690898
HMGCS2	-1.186070529	-5.485097092	-8.223846172	-5.797747529
KIF23	-0.089863294	-0.70749404	-8.22216732	0.139553102
CD74	-1.525148635	-1.829628812	-8.202431355	-1.637483153
MMP7	0.177962763	1.212373298	-8.138502735	0.968002773
JAZF1	-2.694868854	-2.631071222	-8.115306974	-1.533216967
FAM190B	-1.93759976	-4.650449602	-8.111429154	-1.619399137
PLAC8	-0.39843239	-5.602964648	-8.106040809	-3.538510714
SPARCL1	-2.485174689	-5.809878101	-8.092991261	-2.094276086
GOT1L1	0.19689057	-0.645118233	-8.089942635	0.96998028
HBA1	-3.598756363	-6.622965187	-8.08707375	-2.778011921
TSPAN3	-1.761137665	-2.46415869	-8.078752147	-1.998795316

SORCS3	-0.316736307	-0.344603108	-8.044244748	2.881208535
FXYD1	-3.650653401	-6.532714686	-8.001750254	-4.04497822
HNRPK	-2.138401379	-3.226780208	-7.999579548	-0.752146088
LOC729954	-2.426723301	1.877013614	-7.992092998	1.241523424
SFTPA1B	-3.5006929	-7.693908635	-7.967692671	-6.713274607
TAPBP	-2.948003359	-2.591321353	-7.967056876	-0.430045198
TSPAN7	-2.773279112	-5.301209747	-7.95488947	-3.003734542
MTMR14	-1.40928013	-1.596497724	-7.921442581	-0.737811183
CYBRD1	-2.406205292	-5.263117803	-7.896070607	-2.876771047
LOC392871	-0.751931638	-4.217195533	-7.894386982	0.148339284
CA2	0.210676369	-3.608849637	-7.881844203	-4.302519854
GLRX2	-0.544971486	-1.493006511	-7.881560526	-0.54608989
TAP2	-2.017306067	-2.224280292	-7.870876252	-0.200655594
LOC728744	-1.283154034	-2.340638556	-7.85728753	-0.820136411
LOC400759	-3.278542718	-2.753918098	-7.846693437	0.621402661
HLA-DQA1	-0.613600458	-1.089430797	-7.824467369	-1.271755687
PLEKHB2	-1.7532925	-3.450832839	-7.820554575	-2.357190171
CPB2	-5.252437884	-9.253859085	-7.818888214	-6.037692495
CT45A4	1.656391742	-5.838545478	-7.795734862	-2.265077144
PGC	1.002339482	-0.055884482	-7.790394126	-4.867899363
NPNT	-3.506461417	-7.093993232	-7.78070911	-3.745090719
MORN2	-0.650250185	-0.472405511	-7.757052693	-2.726441977
RGS11	-2.283840808	-4.830711036	-7.755475404	-2.471784248
RPS24	-0.757471056	-3.585057119	-7.740009143	-1.232125469
TIMP3	-2.322918992	-5.509567142	-7.723548809	-3.336304276
LOC441019	-1.912285235	-3.15329224	-7.722319644	-0.806532874
CAV1	-2.201590253	-6.572711348	-7.70745259	-4.331013894
BMPR1A	-2.530543976	-4.886372157	-7.701211934	-1.952152005
WNT3A	-5.305581376	-8.136127314	-7.687126289	-6.787439631
OLR1	-1.908118911	-4.38858417	-7.66613925	-2.342483248
LOC641975	-1.620786985	-1.084152553	-7.662614144	0.188372945
ZNF45	-2.224025861	-3.149548334	-7.659906865	-1.776332026
IDO1	-3.37646708	-0.909173589	-7.64213673	2.637536543
IFIT5	-2.346501671	-3.50212774	-7.616839788	-2.707800655
KLHL6	-0.015638971	-3.612168937	-7.612555187	-0.71573049
TRIM37	-1.483246287	-1.876122574	-7.610614432	-1.26084745
LOC644058	-0.42036431	2.541202901	-7.602890776	1.118797431
RTKN2	-2.828965138	-6.712807033	-7.580423515	-4.056102921
NFASC	-2.381753255	-1.126133222	-7.555832128	-0.91114373
SLPI	-1.311498215	-6.567641937	-7.553561157	-1.598829113
SFTPA2	-3.274689142	-8.292543275	-7.52874818	-7.721833513
DIAPH3	-1.41102123	-3.125439186	-7.52053535	0.0377344
HS.434989	-1.263612886	-0.701665497	-7.51792954	-0.645541876
EGFL6	-3.042672689	-4.080952767	-7.490538854	-1.724555908

SEC23IP	-0.815145366	-1.791573125	-7.484463876	-1.650580485
LOC642585	-0.64403595	-1.296674756	-7.479308972	-0.799098139
HS.146882	-1.031803109	-3.477540714	-7.471731987	-0.384364842
WBSCR16	-2.012936403	-2.446393008	-7.453732189	0.014404565
LOC131185	0.0186639	-0.041286642	-7.452484544	0.486814333
FLJ27354	-2.0659246	-4.827368786	-7.443554518	-0.779445906
HS.571297	-1.456520061	-1.588262321	-7.430266964	0.162244123
HS.356079	-2.342349901	-2.37472168	-7.421613528	-1.676142242
RAB43	-1.70870816	-3.357850369	-7.4056486	-1.332076086
FCGR1A	-0.707916861	-9.560289435	-7.404747066	-0.502536991
HS.441076	-0.894358541	-1.679477358	-7.391601415	0.431213973
SELS	-0.394009671	-2.078967937	-7.390026672	-1.755359931
LOC730517	0.123370232	-6.262516961	-7.385324797	-0.373803024
LOC649389	-1.475213241	-0.533658001	-7.381269626	-2.895163011
LOC401286	-6.149479457	-5.245065678	-7.377209852	-6.092700411
CASP1	-1.53253666	-1.901168441	-7.364013751	-1.176847434
C10ORF118	-1.656023717	-2.644305664	-7.357307813	-1.988348019
GSTA2	0.825346815	-1.409749967	-7.353416617	-7.211993599
DCN	-4.079825456	-5.894809834	-7.344656125	-2.675203535
OTUD1	-2.282763387	-4.571830934	-7.341836826	-2.255564096
TEK	-3.149486082	-6.790049157	-7.332053683	-2.497412943
IRS2	-1.838304042	-5.351477952	-7.330024571	-2.220974315
VWA1	-2.632999136	-9.873401944	-7.327909003	0.667487929
LOC100131001	-0.336192802	-1.590756959	-7.308157512	-0.593584129
MYADM	-2.80850106	-6.373020709	-7.300643372	-3.181540711
C20ORF12	-2.637443647	-1.299631237	-7.299803557	-1.735456993
DPPA3	-0.747124846	-0.37706525	-7.278191122	-5.930731824
SYT15	-2.836664508	-2.309123308	-7.277571048	-1.607605269
KCNAB1	-1.293006972	-4.398722705	-7.271033648	-2.99341113
MSLN	0.430556132	-6.554955194	-7.257249842	-2.495625943
MCF2L2	-3.202949728	-1.622231273	-7.250853724	-0.204348761
LOC644544	-3.65530188	-2.106655515	-7.232242929	-2.822908367
ACADVL	-1.984000428	-4.672419114	-7.213617092	-1.144606634
LOC652633	-0.779189327	-0.944790026	-7.201735323	-4.037682308
KIF2A	-1.120358442	-1.092946422	-7.185612191	-0.345419579
SFTPA2B	-2.81034733	-6.131765268	-7.175492139	-5.662125688
CCDC101	-1.305334023	-1.326512435	-7.174217831	-0.762595681
SPARCL1	-2.235579369	-5.592235779	-7.17223506	-2.546561333
ITGA3	-1.864009826	-1.331719484	-7.165741885	-1.346541373
TGFBR2	-1.999981875	-4.772538328	-7.150905146	-2.766832586
MS4A7	-2.383832574	-6.063343547	-7.143855854	-2.875279337
KBTBD10	-1.89316443	-2.884482645	-7.123987362	-1.357908493
FPR3	-0.454942651	-2.881041418	-7.122668955	0.231197295
UQCC	-1.848622719	-1.58247545	-7.11857686	-1.336476025

TMEM16A	-3.964771793	-4.476378353	-7.113483314	-2.293031923
ARAP2	-1.547257473	-2.496474076	-7.107259768	-2.604671075
HS.572246	-1.333211225	-3.787680332	-7.086014673	-0.987117842
LYZ	-0.779285507	-4.598067894	-7.070503245	-0.893940126
SEPP1	-2.689805892	-3.468894331	-7.047651363	-1.869803513
RRAS2	-3.28113542	-3.209350779	-7.043964435	-0.792820884
GSTM1	1.262481495	-1.249558671	-7.03495736	-5.02672962
WIF1	-4.581865462	-6.01561035	-7.025275057	-5.680927795
SMAGP	-0.772473149	-4.293031754	-7.017430519	-3.425253813
ENPP2	-1.789344849	-4.526909446	-7.015852557	-0.877226887
LIPA	-0.457672684	-3.029874362	-7.013005308	-0.858231701
DPYD	-2.604518067	-3.045125377	-7.011897263	-1.455026479
KPNA3	-1.723873202	-4.4433883	-7.00029641	-1.456591594
RCL1	-2.472263323	-3.727284617	-6.998750221	-1.560471136
DBC1	-4.0374634	-0.858021583	-6.985323168	-1.946393539
CFD	-1.760407145	-5.314031186	-6.9810252	-2.485771838
C7	-3.652166093	-6.952263049	-6.970522549	-1.657457221
SLC39A8	-3.048233786	-4.26879231	-6.965864938	-4.032997471
LCN9	-0.086006952	-1.79715408	-6.956481689	0.805125177
FCN3	-2.687590472	-6.381135812	-6.954730048	-3.754043507
CD151	-2.068057486	-3.164996797	-6.93478561	-2.276267846
DSCR3	-1.967615232	-3.606595863	-6.927044031	-1.734651305
C1ORF116	-2.125666259	-1.923009547	-6.926508233	-3.137747118
C3	-1.712170605	-0.614054921	-6.919314458	-1.016550682
FOXD1	-2.480838379	-8.177399518	-6.917227606	6.35282484
CYP4B1	-2.664493111	-4.892946121	-6.915518999	-3.546876725
TALDO1	-5.652588784	-0.694154693	-6.913117989	0.470604976
SLC25A4	-2.023005957	-2.300022628	-6.910652489	-2.914338421
FCHO2	-1.330272608	-2.83144346	-6.902767344	-1.110450674
LOC652815	-2.130172002	-2.783113405	-6.902270449	-1.881666636
LOC648659	-1.481465019	-3.899960535	-6.900227393	-0.442178938
MAP6	-2.618773371	-6.110723941	-6.899341334	-2.781348217
ANKRD37	-1.079074689	-2.702160754	-6.896658746	-2.462958773
VLDLR	-3.326444594	-3.092048263	-6.87690372	-2.26437774
EI24	-2.246027616	-2.995819008	-6.872801294	-1.511041063
TNFSF12	-0.925317522	-2.138913992	-6.870178468	2.098002039
TMEM165	-2.229452672	-2.995579338	-6.86423581	-0.972818696
HSD17B11	-1.655050988	-3.203816544	-6.860340695	-3.150439906
CFC1B	-1.737758289	-2.236159981	-6.849089566	-1.975921693
SULT1A2	-2.161411394	-2.006015177	-6.827663192	-2.1607411
ARSK	-2.746661962	-1.495090857	-6.824001212	-2.978737816
CT45A4	0.831738161	-5.409025627	-6.823918571	-2.280104251
LOC728044	-2.021139938	-0.832937782	-6.821376079	2.583716651
ITFG1	-1.727699435	-2.8727934	-6.820965866	-0.900891417

OR5R1	2.153927022	0.313090286	-6.818363479	-0.001542509
GPR177	-1.372279979	-0.953818682	-6.816294011	-1.333401242
TGFBR2	-2.080933513	-5.131583138	-6.805728461	-2.544316304
CLEC4A	-1.489011267	-2.373214706	-6.798265222	-0.755731752
CST6	-2.100796172	-9.005565692	-6.795247926	-2.240624486
C1QA	-1.93200544	-3.680610443	-6.786829611	-1.325068969
NME7	-2.228100042	-2.492965413	-6.773068285	-1.941457909
IGJ	-3.193282813	1.553346108	-6.771238621	-1.093878233
C2ORF32	-3.226253219	-4.891786467	-6.750545598	-2.011213763
LOC401845	-4.18511254	3.307569147	-6.747221917	-0.004456116
LYVE1	-3.188083358	-7.179925895	-6.746871114	-2.634258557
GPIHBP1	-3.070004631	-8.272173022	-6.739674093	-3.099598917
HOPX	-1.493392524	-6.014448784	-6.738632497	-3.854021478
HPGD	-3.546411365	-6.745419057	-6.736953187	-5.031302554
HS6ST3	-0.405885655	-0.071232306	-6.72945815	0.048920719
GNG10	-1.06305102	-2.749040732	-6.715509895	-1.724825589
TMEM100	-3.981086302	-8.056520314	-6.714430482	-6.22537685
HBB	-2.410490149	-5.404055012	-6.71207092	-3.056082887
C17ORF58	-1.996886132	-2.05014775	-6.708820171	-0.581793938
C1ORF201	-1.788876291	-1.254565665	-6.705722903	-4.070073293
CST5	-1.144454892	-4.62189738	-6.695807669	-5.085106647
COL12A1	-2.855743661	-4.240737304	-6.694752225	-0.90741009
C10ORF73	-2.38997333	-1.512491701	-6.694665494	-2.204351458
C13ORF23	-2.41262738	-3.72449484	-6.669467323	-0.591098857
CYP4B1	-2.16147769	-4.132111705	-6.660433503	-2.746990768
AGER	-3.193582908	-8.195611498	-6.6558329	-5.894560302
UBXN2B	-2.041996048	-2.81450562	-6.655568118	-2.419159931
MGAT3	-2.875933692	-6.956459761	-6.652538581	-4.205710781
LOC100132804	-2.331435494	-3.461051816	-6.651373674	-1.693127481
LOC100133678	-0.639282417	-1.265000104	-6.648314977	-1.104185056
CRTAP	-2.224025063	-3.935147301	-6.640263574	-1.738482448
CYP2B7P1	-3.525725881	-0.255364163	-6.639624851	-2.246751799
LOC653463	-5.883269226	-5.465026119	-6.637455078	-4.362201076
SMPDL3A	-1.426443255	-2.514589856	-6.625760911	-1.51478045
LOC100134563	-0.424945702	-2.829335637	-6.624592777	0.083403804
DUOX1	-2.269704857	-0.034643281	-6.620911644	-3.116351853
NME7	-2.386177675	-2.294507198	-6.615884786	-1.593989923
LOC100131704	-0.608722268	-2.409283795	-6.615608522	-1.019913056
LRRN3	-4.77047369	-6.036591927	-6.591021624	-4.515764686
LOC646201	2.119015365	-2.475117567	-6.578256254	0.609012805
NDUFV3	-1.365609663	-2.905219283	-6.564023858	-1.121880851
LOC730820	-1.365041319	-3.550007916	-6.558408361	-1.652497689
SLK	-2.182883482	-4.453486586	-6.54620552	-1.93680265
CLIC3	-2.310522856	-5.748481594	-6.545893268	-3.3016263

NUDT21	-1.850784328	-2.592995643	-6.544963956	-2.071023975
RPRD1B	-1.972680237	-2.504880264	-6.540617301	-0.772919604
NBLA00301	-1.908975978	-2.171718283	-6.533451655	-1.651779984
RAP1GDS1	-1.847084048	-3.003634681	-6.532771673	-0.616941233
C12ORF40	-1.650858161	0.542554149	-6.531525242	0.453284947
LOC149620	-4.329995308	-0.58891115	-6.526711191	-3.397044398
HBA2	-2.628204049	-5.022235646	-6.523064861	-2.414264987
SCHIP1	-1.937884724	-5.582441238	-6.517625789	-2.658747685
CT45A4	-1.534634789	-5.998844832	-6.512093073	-2.61745304
BCL7B	-1.374473511	-1.096782484	-6.504993239	-1.315922685
LPL	-4.041823327	-6.975151251	-6.498754625	-3.634738384
RNU105C	-2.147993827	-2.375333306	-6.496066299	0.169342933
LOC649555	-1.625159038	-2.913136453	-6.489146652	-2.038243739
GKN2	-0.036861645	-9.457253465	-6.473420451	-4.964164071
HS.333400	0.589937001	-0.079607265	-6.470299429	-0.003126882
LOC729646	-1.206389796	-2.67234747	-6.465325941	-0.975704619
HS.537004	-2.021911321	-3.617654	-6.461211849	-2.146560313
C2ORF83	-1.252521584	-0.859654551	-6.460820955	0.521198652
PQLC3	-1.593649522	-2.911859806	-6.458139907	-1.262740167
MFAP4	-2.851862147	-5.822325789	-6.456057596	-3.553658714
SRGN	-1.920317061	-4.077213786	-6.454811893	-1.613642517
LAMP3	-2.498098018	-3.270320206	-6.45436144	-3.870698981
LOC285501	0.239490949	-2.00354927	-6.452614272	-0.380477885
SLC11A1	-1.833335289	-5.944978706	-6.443943582	-1.985877376
FHL1	-2.867545637	-6.626670878	-6.440297777	-4.250004058
FEZ1	-4.009333132	-5.910675552	-6.439814597	-2.793225327
NBN	-2.897209879	-3.07331972	-6.4250807	-2.934095271
TRIM5	-2.256227121	-2.616126585	-6.422466915	-0.160463225
HS.233165	-0.71193558	-2.507897835	-6.414066358	0.415399336
CCDC132	-2.491795581	-2.786214464	-6.412374841	-0.452693429
FAM50B	-2.188923073	-2.278852384	-6.401667854	-1.924440322
MGAT4A	-1.574030003	-3.450539533	-6.399185427	0.245513366
ARL3	-4.408471269	-9.080397495	-6.391103517	-2.454687943
TXNDC5	-2.377478098	-1.96606684	-6.388613542	0.681683346
IL7R	-2.995419926	-3.585360168	-6.387165137	-1.707254868
PRPF38B	-1.99263538	-2.587624397	-6.38659994	-1.050061101
TLR7	-1.093101169	-4.199616078	-6.384004087	-0.455632852
AGR3	-1.581738626	-3.007420571	-6.383952171	-2.934102733
HS.171169	-1.055305778	-0.305849036	-6.382015192	-1.464109923
FAM107A	-3.24127565	-9.254423081	-6.378830913	-3.946878883
PECR	-2.906780173	-3.662580215	-6.37432983	-2.090593896
LOC643873	-1.580920238	-3.768145411	-6.370178557	-1.885595195
PTPN13	-2.091714052	-1.956058567	-6.370060789	-2.912059751
ZNF555	-0.909668534	-1.126660033	-6.36307906	-0.464483794

IGFBP6	-4.045194772	-4.683035226	-6.362681292	-2.019068685
LOC728843	-1.29678513	-4.357785368	-6.358396297	-1.55539106
LOC100130746	-2.436298058	-3.299009465	-6.351642781	-3.364186657
RTN1	-3.757507965	-3.56635283	-6.350808722	-3.221027862
TMX4	-2.036792219	-1.7249441	-6.344235154	-1.849700177
RASGRF1	-2.482533876	0.416899845	-6.338782463	-2.434270136
HS.583661	-1.525005097	-2.139389238	-6.338617859	-1.542529857
CYFIP1	-1.201863619	-2.951098852	-6.336615699	-1.320922687
LEPREL1	-2.103871263	-3.786367816	-6.3358107	-3.852104258
CHMP5	-1.374640932	-2.941679213	-6.334940271	-2.542318593
AGER	-2.898143292	-7.725187331	-6.331796452	-5.198032537
PGCP	-2.481935615	-2.998844406	-6.316838381	-1.840880736
LOC728782	-1.722104126	-3.930768113	-6.307339752	-1.531491009
IL7R	-3.150427737	-3.876094259	-6.303954713	-1.84191933
HLA-DPA1	-0.831215273	-1.334335505	-6.302360335	-1.102057767
MARCO	-2.84938844	-5.146023402	-6.30000109	-2.562984318
TSC22D1	-2.952060711	-6.285054199	-6.298966352	-2.859996657
LOC100133055	-2.24381507	-2.375469617	-6.27368335	-1.556931265
LACTB	-1.445574341	-2.005056924	-6.269487345	-0.828869127
CD163	-0.920166476	-3.565173657	-6.268741582	-1.179457982
PPP2CB	-3.329613322	-4.770692725	-6.264807537	-2.524620193
LOC100132060	-3.351969722	-4.090885772	-6.260921266	-2.87099218
CCL23	-3.497646997	-5.675604625	-6.254855031	-2.411666126
GPR34	-1.195024337	-3.407978053	-6.248029944	-0.961342346
ANXA3	-2.528629452	-6.421276011	-6.239376057	-3.439896731
TCF21	-3.39640242	-7.333840235	-6.236978551	-4.229944047
SFRS3	-1.906364171	-3.36039246	-6.236931427	-2.274796374
HSD17B11	-2.202905025	-3.049548324	-6.236904857	-2.850821791
XRCC5	-1.46891522	-2.650472384	-6.234833739	-1.325695951
HLA-DRB4	-0.849581819	-1.252177342	-6.233128552	-1.552065601
LRRC59	-0.583644736	-2.090388842	-6.230131594	-0.29343701
RHOT1	-2.388073719	-2.739678088	-6.229302831	-1.943833448
GRIN3B	-1.747758513	-2.790714717	-6.227735382	-4.975820694
SP3	-0.471805334	1.490669508	-6.225502267	-3.807602042
CDC42	-1.882409539	-3.360793259	-6.224485383	-2.965826396
ZDHHC3	-1.038584765	-3.098773519	-6.221347837	-1.024761415
SPOCK2	-2.241786688	-4.766409957	-6.205156334	-2.536725075
REEP1	-2.562063351	-8.132474972	-6.195704372	-1.184763343
FTO	-2.818478186	-2.880889322	-6.18923404	-2.269973144
OAS2	-0.747031134	-2.129856	-6.186873222	-0.936582499
DHRS12	-1.343163108	-1.403504522	-6.180839851	-0.211069971
KLF9	-2.836046453	-5.179704451	-6.171346943	-2.605544085
TGFBR3	-3.115453264	-3.89553637	-6.167153153	-3.5616682
IRX2	-3.036528672	-0.99160296	-6.165920701	-4.699880176

TM6SF1	-1.394447379	-5.85683987	-6.164847066	-1.987195876
LOC389286	1.489947694	1.451985044	-6.157905451	-0.041724471
FNTA	-2.038624198	-3.262082861	-6.148241189	-1.409854592
KITLG	-2.871878801	-4.514160256	-6.142874513	-2.379516322
SFTPB	-1.43829119	-2.263346679	-6.139124571	-3.028713175
LOC643778	-3.070542765	-6.544638887	-6.138613835	-2.26375535
RTKN	-1.122660317	0.757001241	-6.133391079	1.412590119
OSCAR	-0.230596571	-3.827618672	-6.126718102	-2.369248227
ICAM4	-2.442315733	-4.281324301	-6.122688631	-2.623585478
EPC1	-1.897635804	-3.436230387	-6.118718404	-2.369139932
LOC255275	1.029768554	-2.828152673	-6.114267221	0.635375418
CBR4	-2.388630785	-2.486039262	-6.112383601	-1.080754494
FOXF1	-3.438819062	-6.730693112	-6.110893707	-3.7242124
RNF144B	-1.843684977	-3.868825732	-6.101932353	-2.126736259
AKAP11	-2.557993262	-4.841797279	-6.100287857	-1.661219739
FAM150B	-2.64446513	-6.320483871	-6.099017411	-3.354007283
COBL	-2.552765978	-3.268574525	-6.095920939	-2.28150186
FLJ37453	-3.356800153	-1.48959873	-6.092384884	0.558824078
APP	-2.063521457	-3.38021183	-6.088573893	-2.102271034
MYLK	-3.459992125	-5.97944347	-6.08114633	-2.895397101
HS.539195	-0.40779861	3.394467112	-6.077389651	-0.563123805
VTA1	-2.639409831	-3.226197824	-6.072865085	-1.571270779
LOC100128016	-1.314893365	-2.374785356	-6.070286877	-1.060924652
MAPKAP1	-1.822268171	-2.816673088	-6.065009933	-1.754785986
CHPT1	-2.790116869	-4.198885257	-6.059939088	-3.268767762
ALOX5AP	-1.984380158	-3.158701624	-6.058326226	-1.996847621
MAOA	-2.613561233	-2.917981439	-6.056934001	-3.560522523
NIPSNAP3A	-1.869755946	-3.182061417	-6.056834343	-1.949606455
RSC1A1	-1.601981813	-2.551806874	-6.044422695	-1.748277753
LOC727821	-1.787342722	-3.614103861	-6.042924645	-1.525138831
CYBB	-1.915766675	-3.074846661	-6.0322945	-0.911195171
HS.551438	-2.818884308	-2.593927626	-6.028415274	-0.461676754
C21ORF37	-1.452740065	-2.651509232	-6.028069125	-0.423046863
ITM2B	-1.47816874	-3.357930832	-6.027572824	-1.621132562
TMEM77	-1.123694666	-2.647188118	-6.021980567	-1.520155596
HS.241559	-1.634673609	0.447230906	-6.02053375	1.688013267
NME2	0.997716291	0.770883505	-6.019894562	3.877799113
ARRDC2	-2.078994327	-2.375530327	-6.01587784	-1.105141924
WRB	-2.947773559	-3.22508104	-6.007201529	-1.922343451
DNM1L	-1.880276439	-3.503756414	-6.006772911	-1.399402743
MYH11	-2.61050757	-5.568142402	-6.004879579	-3.732466513
CITED2	-2.893518261	-4.495443231	-6.002464526	-2.128915599
TMPRSS3	0.651795059	-0.667467646	-6.000110225	-0.461932644
HS.133009	-3.325069898	-2.266881209	-5.997556222	-0.40261177

C14ORF144	-0.385565214	1.340461213	-5.996942388	-1.162312603
SFT2D3	-2.24132844	-3.336444835	-5.996287358	-2.217233915
SFTPC	-0.88182045	-0.675812566	-5.992488007	-4.393982097
CCDC8	-1.523121181	-1.522378584	-5.985327563	-3.542776142
ZFAND6	-1.603779616	-3.18889466	-5.980394288	-2.735348943
PPP2R2D	-3.078466765	-3.818354438	-5.972609383	-1.400029758
ADRB1	-5.430348654	-5.043683207	-5.972247511	-3.069132603
HS.496187	0.065827778	-0.137734903	-5.972114431	-1.818561873
ATP2C1	-2.688444453	-3.585015387	-5.971324726	-1.667425775
KIF18B	0.46308021	-1.402706056	-5.969761782	0.365750743
LOC727900	-1.825076837	-2.274026272	-5.967010336	-1.803553537
HS.545519	-1.730521518	-1.794366733	-5.966659609	0.037920217
SCGB3A1	-1.13507143	-1.609368538	-5.961820552	-3.469540347
MRE11A	-0.780802911	-1.587420071	-5.960424463	-0.111439522
SFTA2	-2.137900887	-1.543842422	-5.956180717	-3.334727869
LOC728216	-3.327627344	-0.821098334	-5.952155331	0.064062275
TMSB4X	-2.161481551	-3.25588904	-5.948267449	-1.480268247
XKRY	1.135251635	-3.016177837	-5.935960922	-0.889152505
PSMD6	-1.708624488	-2.997409952	-5.93203698	-1.83041803
MAD2L1BP	-1.278564516	-1.355716292	-5.924395144	-1.093273314
LOC649839	-1.621251873	-2.602934971	-5.917843308	-0.307387568
C1ORF41	-1.206996785	-0.666744699	-5.91189835	-0.548680332
NCKAP1	-2.028511744	-3.665169681	-5.906895346	-2.212515011
LOC390748	-0.524843777	3.923173169	-5.905848799	-0.001455291
SFTA1P	-1.846479808	-3.557386716	-5.902697361	-4.235786639
SRI	-1.294193534	-1.377020102	-5.901047733	-1.660910841
ZNF366	-3.059020357	-5.837951681	-5.900339756	-1.638554452
TGFA	-1.432994601	-2.574216932	-5.896864634	-0.831143112
THOC3	-1.828138953	-1.624349325	-5.896472011	-0.489329322
CA4	-2.75171533	-6.906451089	-5.895077575	-6.003397605
MMRN1	-2.405617882	-6.011800361	-5.89308858	-1.869592514
PPAP2B	-2.412301924	-4.433035115	-5.891230127	-2.671556481
LOC730415	-0.557558529	-0.944602192	-5.888871915	-1.602846111
CSNK2A1	-1.205077938	-1.94997717	-5.876680383	-0.61002109
LOC645626	-0.474034891	-0.995812655	-5.873033337	-1.172391803
SERPINA1	-1.243156943	-5.056454796	-5.871255337	-2.457322966
CLEC12A	-1.779251971	-4.344067232	-5.859974254	-1.515551392
MASTL	-1.368055085	-1.399333896	-5.854522883	0.184573474
ALOX5	-2.107899557	-3.323332726	-5.852442582	-2.230385618
CENTB2	-2.153637557	-3.239231311	-5.844157186	-1.393312216
HBEGF	-1.632726979	-5.756640332	-5.844022343	-3.257827096
TLR8	-3.003108473	-4.500979556	-5.83240587	-1.087221742
NOL4	-1.261783276	-0.097018061	-5.830682345	-0.80415071
WDR16	-0.693556298	-3.579354424	-5.824149882	-5.53430364

LOC653082	-1.282143922	-0.128108603	-5.823160674	-2.575327303
RNF8	-1.351833659	-3.590041946	-5.810974197	-1.100056197
B2M	-0.011561105	-1.200597671	-5.809577109	-0.699667896
TBC1D9	-2.744680742	-4.229361696	-5.808659788	-1.310126509
BCKDHB	-1.926767477	-2.103323894	-5.805655455	0.022114807
SNX24	-2.776801896	-2.995640503	-5.80543368	-1.057477988
LOC645946	0.43313705	3.907492994	-5.804941614	-0.619206807
P2RX2	-3.656066233	-3.208766367	-5.803840533	0.159353484
GATA3	-1.849105166	-1.822420345	-5.803452661	0.023498029
LOC441073	-1.985793221	-3.383169329	-5.801267432	-0.69605101
HS.105102	-0.763725767	0.833824355	-5.79527886	1.382697448
DNAI2	0.192547274	1.974532995	-5.792316228	-3.098184464
HLA-DRA	-0.591958966	-1.150264825	-5.79227036	-1.288168615
OR5L2	-0.032698005	0.209504674	-5.792126175	0.549785162
PLK2	-1.747270779	-3.368320338	-5.790076818	-1.321172805
ETFB	-0.743657235	-1.614428947	-5.78323595	-1.695815735
VAMP7	-2.140146173	-3.146402226	-5.782487011	-1.307605946
LOC128192	-1.697195642	-2.835927779	-5.78037246	-0.784389016
SHROOM3	-2.279975538	-4.651800744	-5.779621291	-3.555684574
C14ORF78	-2.319252544	-1.131069292	-5.776250293	0.431046716
GDF10	-4.376325097	-5.936592082	-5.774367539	-4.875456937
LOC439994	-2.666158813	-6.296722664	-5.773672994	-0.561911001
UBXN11	-0.164209106	-1.772033305	-5.768529116	-2.025078363
CRYAB	-3.48417333	-5.411516617	-5.759337626	-3.942907436
APOBEC3G	-1.666200561	-1.604734785	-5.751445684	-0.046249119
IP6K2	-1.136731887	-1.650945325	-5.733182407	-1.387444505
EIF1AX	-3.112803075	-3.672416337	-5.730295114	-1.660251075
CD97	-2.202140449	-3.734278129	-5.73015524	-2.04777939
SLCO2B1	-0.928767052	-4.118447046	-5.729191147	-0.899138684
CD2AP	-1.138103149	-3.004912645	-5.72708013	-1.955016942
TMEM188	-2.397651965	-3.357428703	-5.722010486	-0.76873642
ING1	-2.369668488	-3.759859153	-5.716949327	-1.799444695
HS.573763	-0.777442267	0.775077378	-5.715372796	2.768820797
DNAJB4	-3.28416021	-6.066659654	-5.714089534	-2.187594868
S100A4	-2.253598585	-3.774682123	-5.71372842	-2.260860129
ENPP2	-1.53909331	-4.783895842	-5.708458912	-0.988414849
TXNDC2	-0.173820267	-0.161528838	-5.704647664	0.036856653
ADORA2B	-1.147459338	-1.397970436	-5.700873373	-0.969031872
ARF4	-0.554771673	-2.549976256	-5.697093151	-2.186378235
SHE	-2.739062454	-5.740644682	-5.694299945	-2.746826905
FCER1A	-1.826122708	-3.069074941	-5.691236103	-3.810390273
SDHD	-2.407007309	-3.69959132	-5.68948361	-1.687538305
GIMAP7	-3.826106978	-7.83352435	-5.689069842	-0.794735744
FMO3	-3.208397807	-4.468614893	-5.686602267	-2.161238662

TSC22D3	-2.156731683	-3.751087835	-5.686356487	-1.759170227
HIGD1B	-2.737467845	-6.743213684	-5.685500399	-3.943856558
CEBPA	-1.102647193	-1.831128077	-5.680338674	-0.512282187
CTSH	-2.09362225	-0.282262711	-5.675402167	-2.181199861
UTP14C	-2.595979148	-4.859743399	-5.674777427	-1.35949864
ANKAR	-1.626737863	-2.336549746	-5.67377932	-1.780731328
MSRB3	-4.048374928	-7.040246868	-5.670260256	-2.697072532
AHCYL2	-1.527513318	-0.138823348	-5.664164493	-2.739763829
CD93	-2.773470453	-5.614548973	-5.663422155	-1.626844244
KIF21B	-3.242014761	-1.968464606	-5.662782482	0.169899443
SRGN	-1.582728091	-3.649447739	-5.65494633	-1.596947324
ALPL	-1.574602424	-5.240650922	-5.649105909	-2.752268907
LOC650095	-0.616883754	-1.468131648	-5.646641307	0.583915984
CALCRL	-4.062581139	-7.225861869	-5.646230907	-3.021551955
RNASE9	-0.706014242	-6.448537126	-5.644409861	-0.981073653
VWA3B	-1.66454803	0.029108293	-5.643874724	-3.179629297
HLA-DRB6	-0.826544173	-0.782003506	-5.643168709	-1.765553374
SNORD113-1	0.824205315	0.249370499	-5.642997773	2.711108726
TYROBP	-0.253254999	-2.700201164	-5.639850713	-1.107304372
PGAM1	-1.479696614	-2.9684681	-5.639758546	-1.008704812
ST7	-2.34266112	-1.24905911	-5.63958858	-1.938117345
CREG1	-0.819757934	-1.389182667	-5.638643618	-0.958504975
UBL3	-2.053807033	-4.798123744	-5.63627298	-2.640656808
STAC3	-2.509439307	-3.730197474	-5.633606325	-0.21118312
LOC285016	-2.281881543	-11.57193393	-5.633421287	-5.946305461
BMP6	-4.616812062	-8.434482188	-5.632824755	-3.044545748
BTBD3	-1.818050301	-4.10858279	-5.626954023	-2.217697807
PRMT3	-2.138309847	-2.046835486	-5.621983059	-0.148229416
BAI1	-1.849943904	-3.427692786	-5.620789688	-1.78290107
VILL	2.407106697	4.09414853	-5.616400747	-0.747376025
LOC652614	-1.40191265	0.044731965	-5.614830978	-0.777894957
HS.566669	0.459736268	-0.831219829	-5.608310099	1.065197473
LSM8	-2.837617159	-3.915034426	-5.607576571	-0.996982716
MTUS1	-1.582567039	-2.429542822	-5.603394026	-1.790414903
CCL14	-1.497342354	-5.723228086	-5.598048667	-1.080685063
BBS9	-1.23768566	-1.292800917	-5.597542805	-1.720209261
LOC441481	-0.137255051	-1.909792718	-5.59452091	-0.153315765
SLC46A2	-2.697568456	-6.492476229	-5.590218217	-3.390036173
IRS1	-2.934519381	-2.984235828	-5.588983642	-3.314908033
HLA-DRB4	-1.698919614	-1.654565713	-5.582913969	-1.921519375
GBP4	-3.212238146	-3.083065172	-5.581852971	-0.848577177
C1ORF116	-2.407640005	-4.019762264	-5.580475935	-3.249479145
PDIA6	0.355499529	-0.895999866	-5.577484974	1.290071404
EFCAB1	-1.38279748	-2.793413179	-5.573464129	-6.228181194

CXCL12	-3.253090807	-5.986445807	-5.572410467	-1.556052652
C4ORF31	-2.913310314	-4.368774746	-5.571855169	-3.201874089
SIK1	-0.252546943	-5.21176493	-5.562243713	-0.953453572
C19ORF33	-0.029690504	-3.203926186	-5.562125671	-2.181987743
KLC4	-1.068364959	-1.443370145	-5.55705545	0.347289022
CD97	-1.888023445	-3.428508992	-5.550994004	-2.040826492
LOC399942	-1.068257223	-2.925353145	-5.54368919	-2.083407611
NR4A2	-1.869827821	-6.618266811	-5.543524401	-1.321086726
CD86	-0.823446537	-3.62649195	-5.539590505	-2.699763385
TMEM17	-1.433406711	-2.916707542	-5.536242496	-2.603687994
LOC440928	-0.567714938	-3.061688055	-5.531763779	-3.831950403
PPM1D	-2.554259918	-3.877363996	-5.531266006	-2.249898182
DEFB128	-1.109298338	-0.543917183	-5.529394821	-0.209216817
CPVL	-1.171724958	-1.083014827	-5.528507852	-1.113426885
DCTD	-1.168434439	-2.252225462	-5.528091733	-1.223354619
APP	-1.353333341	-2.526120452	-5.527963813	-2.001997763
44089	-1.430270905	-3.068138411	-5.527446007	-1.36325868
CPNE4	-0.519981373	-0.639619592	-5.52546296	0.527898366
IL15	-2.679774069	-2.271459096	-5.524710331	-0.700593824
HS.62314	-2.279929746	-4.361050027	-5.52393251	-1.758484489
EPAS1	-2.556545719	-5.401628769	-5.523820084	-3.235733567
RAB43	-2.950715605	-1.765277922	-5.521864194	-0.854244895
LOC100129773	-0.463885264	0.355563905	-5.519723967	-0.513521066
CDK6	-2.224221639	-5.530473511	-5.51804945	-1.310481689
PPARG	-2.162921806	-6.213005412	-5.517810588	-2.717548705
PLGLA1	-4.171562907	-7.81972204	-5.514830838	-1.682879066
PORCN	-0.993102478	-0.418812854	-5.511559342	-0.749011563
GK	-2.022883242	-1.47518968	-5.51148608	-1.18388105
CH25H	-3.17567664	-5.085298595	-5.511442192	0.343301364
CLEC2B	-1.64381689	-3.990742297	-5.510823247	-1.498606879
FBLN1	-2.120223817	-4.078352863	-5.509414265	-1.108789425
DKFZP779M065	-2.358952102	-1.38776498	-5.509373212	-1.104271866
LOC389156	-1.994798498	-3.938909648	-5.506624245	-1.449871215
ZNF580	-1.414773103	-1.838684918	-5.506017265	-0.026362951
UBE2W	-3.779600016	-3.033524553	-5.505276046	3.918090584
LOC100130511	-0.647814565	-1.759938776	-5.50320987	-0.79918661
CD52	-0.048477159	-2.518594012	-5.50052344	-2.086813724
C11ORF73	-3.784192158	-2.968066047	-5.496629579	-0.796874155
RNASEH2C	-2.34232937	-0.342729642	-5.494195357	-0.868448044
LOC100127894	-0.534254014	-1.404647446	-5.493615531	0.33494319
ACOT9	-1.248725547	-2.295699749	-5.492185059	-1.390253013
LOC648405	-4.554359053	-4.781006694	-5.489488519	-0.445215004
FLJ21986	-3.028196715	-5.594568071	-5.488833412	-3.027295146
CASP1	-1.883450559	-1.91839897	-5.480059997	-1.052868052

TMEM209	-2.52446471	-1.977007831	-5.476993892	-1.211914082
LOC100130351	-0.737949408	-0.054251185	-5.476803229	8.782526469
S100A4	-2.217399757	-4.043170726	-5.470414093	-2.581823275
KRTAP10-8	-0.360079786	-1.423058612	-5.469857226	0.190553779
MS4A15	-4.2932982	-6.100534232	-5.467791351	-7.595958439
C1QB	-1.316209804	-3.173267408	-5.466177179	-0.761533381
LST1	-0.809083002	-1.429139095	-5.462762348	-1.668451815
C13ORF1	-3.193870962	-5.060401302	-5.462064385	-2.479753543
CTSH	-1.046429943	-0.8167982	-5.456431857	-1.872247633
ZAN	-1.939291318	0.300654208	-5.45588199	-0.740341652
COL4A4	-3.23543992	-4.53924906	-5.451393936	-2.352271993
FMO3	-3.859436163	-6.007698862	-5.44826672	-1.677204874
HBA2	-2.748729977	-5.094253083	-5.448216188	-2.512549356
ALAS1	-2.18279216	-2.832177196	-5.445312583	-0.974867101
CD47	-2.885772521	-3.881954646	-5.443576063	-2.80714567
EXOC6B	1.426574121	-1.827820802	-5.441737785	-0.81259301
CAV2	-2.980032715	-5.463463217	-5.441533938	-3.668081957
CRK	-2.681306523	-3.648222982	-5.440855089	-1.579008464
CYB5A	-1.79629496	-3.512890605	-5.438906075	-3.115831669
TMTC2	-1.354255579	-3.171977314	-5.434669547	-2.566963634
GPR160	-2.767757901	-1.951544373	-5.433260228	-0.776251374
ANO7	-2.015212421	-0.560554562	-5.42944064	1.593078834
TM4SF18	-1.725140195	-6.590408557	-5.425554803	-0.452843719
HLA-A29.1	-1.723926429	-1.495990729	-5.424847269	-0.059092553
SERTAD3	-1.134387015	-3.03532844	-5.421597072	-3.019304579
MGC70870	0.561533267	0.051131641	-5.421128305	-0.381377075
LOC344405	-2.666398214	-2.429411907	-5.419338677	0.724880444
LOC647797	-0.012105705	-1.3914486	-5.419186119	-3.219970039
EDEM1	-1.844939061	-3.114851559	-5.418812917	-1.686510877
PTGDS	-2.488262309	-4.581708115	-5.416030456	-1.857245823
MRPL20	-1.15472683	-1.672216515	-5.415307751	-1.586671368
HS.541315	-1.029488506	-1.964146726	-5.414797922	-0.552287996
EPHA2	-0.780093941	-4.207134055	-5.41200482	-2.409336599
TMEM41A	-1.692480969	-1.704768214	-5.405344156	-1.678831485
LOC648024	-0.811098173	-3.486921805	-5.403271752	-0.901574563
DLEC1	-1.33825821	-1.783492989	-5.398474116	-3.390146318
CAT	-3.34755876	-3.381798243	-5.3983614	-2.897038551
ABCB1	-2.245246569	-4.611726905	-5.397215849	0.405601932
RAB11FIP2	-3.093621505	-2.777165557	-5.396446253	-1.394069816
THSD1	-1.220186649	-5.303716764	-5.396254991	-1.292522186
KAT2B	-2.854704594	-2.680622687	-5.395159305	-2.389823729
ACVRL1	-2.859090234	-6.215933852	-5.393355454	-2.743674799
LAIR1	-0.583817561	-2.958236764	-5.390567308	0.286023956
PLS3	-2.623222754	-4.335584533	-5.388906644	-2.212154923

DLG3	-1.786477032	-0.634031305	-5.38792066	-0.214139068
TMEM225	-0.51982866	-0.690196587	-5.383138382	-0.160793389
LRRN2	-0.801772583	-3.992677102	-5.375896084	-0.27104306
43895	-1.319910716	-3.878424956	-5.37067587	-0.572320997
HS.541440	-1.333493613	-1.093137936	-5.368835243	1.235271283
C9ORF24	-0.350541271	-2.439843844	-5.367950517	-7.318621787
OSCAR	-0.555745548	-4.217863653	-5.367422315	-2.084972791
F2R	-2.325761886	-4.249641764	-5.361046735	-2.174052492
LOC402644	-1.813535964	-2.886163943	-5.360816925	-1.067783141
PDE7A	-2.365005046	-2.64451189	-5.359319507	0.167713571
RNASE4	-1.398406748	-3.20830331	-5.358221061	-3.579636199
SLBP	-1.856490738	-2.078303342	-5.34748266	-1.146933239
MIR187	-3.370431276	0.92207457	-5.339656437	-1.084678309
TP53AIP1	-0.426631258	-3.092088753	-5.338939483	-0.977750331
DNAJB6	-1.385527465	-2.823254096	-5.336527094	-1.798249249
FAS	-1.252247219	-2.611103507	-5.334365556	-1.28003455
EEF1B2	-1.24121996	-3.8552666	-5.33070937	-2.54055365
LOC641849	-1.300340262	-4.296036324	-5.329398493	-0.844053403
FMO2	-3.14111223	-5.830459873	-5.328930606	-4.490834994
ID2	-3.031171005	-4.798364134	-5.328431626	-0.975760836
C13ORF15	-1.676272497	-4.967533435	-5.327183773	-2.951569189
GRP	-2.297126684	-2.309606675	-5.326563266	-3.611408487
CFTR	-0.920643471	-1.459010497	-5.326474589	-1.622341118
INMT	-3.247499249	-6.37333764	-5.321862411	-4.671972073
CASP7	-0.727326956	-1.453707332	-5.319130884	-0.664829183
NEDD9	-2.851647449	-5.095187888	-5.31520703	-2.779722379
TSPAN13	-2.008101865	-2.173775167	-5.313698629	-2.803343261
LOC643870	-0.166922254	-3.53306217	-5.31044907	-1.28301204
DOK1	-1.200797852	-0.691281126	-5.30932287	-1.026335105
ZBTB16	-2.8225623	-8.105063409	-5.303384476	-3.567224086
PGM5	-3.448587473	-2.50079097	-5.300888072	-3.691954295
FAM47B	-1.106512402	1.077563516	-5.297555155	0.748523134
HS.565303	-4.214960293	-1.623751459	-5.297443344	-1.454737258
METTL7A	-2.718042376	-4.693898571	-5.294582392	-2.741821057
CASRL1	1.485411042	1.568270153	-5.291737836	-0.889937888
EIF1B	-2.084557064	-3.328271487	-5.291618179	-1.894833041
CYBRD1	-2.634782763	-5.344814127	-5.290765328	-2.79476106
CXORF56	-2.277645619	-2.619811855	-5.290310472	-2.03658096
VRK2	-2.031151242	-1.582252201	-5.288345515	-0.362414107
HS.578738	-2.298229955	-0.72554957	-5.282306352	1.610439798
FRMD4A	-2.598079694	-5.522494404	-5.28087312	-1.541290928
LOC653879	-1.304688761	-0.745891631	-5.27934471	-0.811851522
MMEL1	0.91160302	-1.648778123	-5.278189257	0.66646527
TMEM98	-1.443125312	-0.819681489	-5.277259742	-1.424067983

CTSC	-1.430620103	-1.598875571	-5.277064875	-0.205250633
RBM4	-1.616639327	-1.858287044	-5.276096967	-1.154426637
TADA3	-1.070240257	-1.754900945	-5.275262586	-1.159911038
MAP3K8	-2.499702257	-4.44933064	-5.274220764	-0.873277652
TNFSF13	-0.996088322	-4.762126112	-5.271381037	-2.448654219
PLAUR	-0.171198231	-3.308078784	-5.266418002	-0.817844864
SCEL	-3.11051694	-4.176798034	-5.264828357	-1.693697776
C6ORF25	-0.577745406	-1.306150279	-5.264267388	-0.552030825
APOLD1	-2.390442165	-5.673840312	-5.263152024	-0.654679563
FLJ35880	-3.222277962	-3.582920877	-5.262705711	-2.121353551
LOC648138	-1.601170418	0.430337785	-5.260885173	1.002325104
AP1S1	-0.815468798	-1.580049423	-5.259118186	-0.561871305
STARD3NL	-1.93151811	-2.935063895	-5.255950561	-2.822485132
DUSP1	-1.568908204	-5.356353268	-5.255154406	-2.084323413
C14ORF149	-3.41587515	-2.951369453	-5.253818803	-3.422090759
RBL2	-1.600492094	-2.429207699	-5.248448498	-1.385364529
RAD18	-0.393687218	-1.544834863	-5.247204163	0.738710782
CDV3	-1.565297213	-3.220933937	-5.246915986	-1.303022848
LMO2	-2.415462649	-4.403202973	-5.246796292	-2.116838381
SPTBN1	-3.025068954	-6.435690195	-5.245754616	-2.878698047
NSF	-0.958166986	-1.458056467	-5.243643368	-0.559641819
ACP1	-1.131284511	-2.448550478	-5.236659521	-1.381782978
PGM5	-3.572582193	-2.269258285	-5.236428316	-3.451903918
SERPINA3	-0.910293415	-5.210832544	-5.236282702	-1.236301563
CCDC89	-0.778903312	-3.250021566	-5.232169257	-2.831210156
VPS24	-1.98236585	-2.687439021	-5.228292299	-1.723493207
ADHFE1	-2.264442044	-3.438060477	-5.228108201	-0.607105277
HS.574769	-1.149949546	-0.222321282	-5.226716943	-0.113041534
HS.324250	-2.307163527	-2.065605021	-5.226658332	-2.44701037
GPR177	-2.287663765	-1.820612957	-5.214344513	-1.423964032
LOC100130221	-0.069594863	0.198125049	-5.214335899	2.202278597
AFF1	-1.957935393	-2.652821551	-5.213088838	-1.114407282
CAV1	-3.587900072	-7.549852457	-5.210510172	-4.488153027
ITM2A	-2.236271865	-4.456312253	-5.210109749	-1.690986058
PTPN2	-1.673699097	-3.162806232	-5.209267862	-1.475950301
HS.466840	-1.474869454	-1.556855881	-5.208944799	0.807823237
TCEAL2	-3.511733855	-5.530629977	-5.206312785	-5.206398342
STARD7	-2.082099952	-3.676295556	-5.203701732	-2.078487079
C10ORF75	-0.875085672	-1.119593687	-5.201212167	-0.054209109
TPK1	-1.582984513	-3.201366136	-5.198795581	-1.486120067
MMP28	-1.698661497	-2.916542339	-5.198336134	-2.443160609
CACNG1	-2.898182071	-2.071673489	-5.194270312	-3.682870322
GPR27	-1.802948678	-3.528047743	-5.193879432	-1.003321788
NDRG2	-1.133625754	-3.097221275	-5.192014961	-2.192410445

FKBP1A	-1.276687156	-3.296374904	-5.18837823	-1.541748479
LOC389156	-2.089049874	-3.936445938	-5.182985414	-1.470220997
KRTAP10-4	0.506677638	-1.338567494	-5.179555119	5.007050478
LUM	-1.809032498	-3.017249651	-5.173922045	-1.040308372
MYH16	-1.703214997	-2.934429985	-5.173352284	2.779556909
AHNAK	-1.817933795	-5.366676687	-5.170201743	-2.847362515
EYA2	1.864466266	-1.041683041	-5.170008935	1.12710328
CD300LF	-0.752065195	-3.079365092	-5.169813302	-1.253763514
NUDT1	-1.203822684	-1.998666325	-5.166797479	0.754332498
TSPAN4	-1.718972769	-3.857739032	-5.166198263	-1.458260205
TMEM140	-1.54262777	-2.435402982	-5.166084953	-1.318127912
USP48	-2.706909805	-3.663648	-5.154755633	-0.526836091
CLEC3B	-3.171957847	-6.755285598	-5.153975456	-4.191728244
RNASE1	-2.353121016	-0.418968929	-5.153547995	-1.858642035
LOC728820	-1.907022018	-3.671374694	-5.152071834	-0.771462377
FILIP1L	-0.392062476	-0.985258202	-5.152018114	0.507492163
KRT76	-0.676485853	0.812965964	-5.151825664	-0.807913438
LOC643668	-1.675645148	-2.478568769	-5.149618144	-1.009385436
HLA-DRB1	-1.785278783	-1.318668787	-5.149343495	-1.24691722
TPSAB1	-2.862564808	-2.915483263	-5.147955303	-2.349762364
RELL1	-2.529835192	-4.600189865	-5.144576492	-2.046415099
LOC100133583	-1.460703951	-1.178855786	-5.143852073	-0.726534847
RPL21	-1.517222578	-3.601437002	-5.143661659	-1.229042752
AP3M1	-1.938097732	-3.379392548	-5.142240148	-1.623272428
EBPL	-1.611881681	-3.354236047	-5.141706235	-1.656154694
FXYD6	-2.454650551	-6.838851682	-5.141459285	-2.473293057
C2ORF40	-2.355988496	-6.815469037	-5.140538137	-6.192279884
SGCA	-3.179770989	-5.18878531	-5.13954944	-1.962717219
LOC100131205	-1.532097818	-3.53586385	-5.136384979	-1.194405043
DGKH	-1.754147737	-2.314193197	-5.133979397	-3.420713973
PPP1CB	-2.296705921	-3.260045196	-5.13286158	-1.666410743
HS.25151	-2.634918651	-2.294034324	-5.1305445	1.791993882
OAT	-1.107696	-2.594794131	-5.130276063	-1.079791695
ANXA1	-2.046476037	-4.036615787	-5.125212339	-2.239627973
FAM162B	-3.330862023	-5.69079118	-5.124005642	-3.727856267
LYRM4	-1.227302735	-2.449845089	-5.122633596	-0.754843142
SMC3	-1.512040723	-3.130264851	-5.122539824	-1.610110221
FAM90A19	2.159382155	-0.495127902	-5.122270559	2.138767338
KLRF1	-2.786045852	-4.92589995	-5.121325336	-1.238005568
IQGAP1	-1.498579627	-2.810026236	-5.120798117	-1.427178085
RASD1	-2.935746323	-5.62197855	-5.119113186	0.620077779
SLC34A2	-3.636847494	-2.974945256	-5.118292653	-1.909936344
HERPUD1	-1.151659638	-2.668607602	-5.117030893	-0.655492299
MT1E	-1.619749338	-2.85209696	-5.114975291	-1.986990319

RCBTB2	-1.871614009	-4.391722682	-5.112778544	-1.12493493
CD302	-3.036199754	-3.953780012	-5.11057694	-2.994225939
UNC13A	-5.377997858	-1.248007359	-5.10330155	1.108933357
LOC727848	-1.019493523	-1.605444116	-5.102981972	-0.860266464
CXCL12	-4.146433518	-6.781694012	-5.091040746	-1.999259096
TMEM16B	-2.874960331	-4.790023635	-5.090764727	-1.966235407
MLL3	-2.50204873	-2.590977632	-5.089740986	-0.058733329
LOC652846	-2.570753017	-5.575393772	-5.087837489	-5.758287621
LOC340274	-0.934824299	-3.866149475	-5.086927358	-0.437183824
COPS4	-1.98130608	-3.363076046	-5.086477217	-1.631637435
LOC730360	-2.224750653	-0.757700718	-5.086005427	0.370941884
PLLP	-0.662317331	-2.774694992	-5.085835714	-3.261018827
HS.540005	-0.450764194	-1.193746027	-5.083949084	-1.558676039
SMAD6	-3.860864771	-5.48398741	-5.083866974	-2.844534678
TPSB2	-2.825154536	-3.362973048	-5.079631813	-2.466096718
C17ORF91	-2.432481195	-5.112433458	-5.079488537	-2.249127863
LOC440349	-0.264723423	-1.804290205	-5.07920911	1.323615863
DUOX1	-1.642196737	-0.014605178	-5.078777463	-3.848294003
C2	-1.865989393	-0.011261776	-5.078295989	0.490669309
AQP1	-1.679193382	-5.937079746	-5.076531542	-2.600777394
NRBF2	-1.824745868	-3.872913366	-5.076524353	-0.719996971
GCH1	-2.550503661	-2.563312201	-5.073273951	-0.35169504
TMEM191B	-2.996929174	-1.597901742	-5.073070886	-0.356864232
ZNF550	-2.469989987	-0.713551711	-5.068379353	-1.512719978
CD55	-2.107581455	-5.681149321	-5.068099251	-2.880542289
TSC22D1	-1.848828769	-4.592421162	-5.067970281	-2.518846131
EIF4E3	-2.996796089	-3.537889777	-5.067763117	-1.494884144
PPP1R1B	-1.338603145	-0.296627225	-5.067686892	1.956429175
ECE1	-2.538198021	-3.951611394	-5.067624952	-0.406689194
GZMA	-2.417979216	-2.758414724	-5.067082589	-0.129852586
TADA1L	-2.40452994	-2.121683961	-5.061246807	-0.863231371
HNRNPUL1	-1.351371095	-2.122211096	-5.06068672	0.26044045
KIF11	-1.184131233	-0.979887379	-5.058199192	2.116331973
HS.571950	-0.781929567	-0.190207563	-5.056084714	0.235326286
TMEM18	-1.952165356	-3.167476941	-5.055248047	-1.507580035
GSTM1	-1.303886841	-1.10949059	-5.053431321	-1.150085518
APPBP2	-3.182193902	-2.002164274	-5.0533583	-1.412073109
GPNMB	0.549111132	-1.492017962	-5.052817112	-0.238112165
SNTB2	-1.996551637	-4.77814749	-5.050500808	-1.143169215
GPAM	-2.877152445	-2.290156837	-5.048166278	-1.808273711
LOC728666	-0.877795536	-1.806720745	-5.04781691	-0.613648515
PPP4R4	-3.016718949	-5.112679196	-5.044969843	-3.55784648
MGP	-1.53298989	-3.306763912	-5.039024653	-1.938403095
FLJ35801	-2.762950817	-3.586486142	-5.038492444	-1.565478093

HS.560343	-2.927438634	-3.093289873	-5.035390096	-1.311393943
RERG	-3.452940781	-6.713419277	-5.032639917	-3.717888098
HS.571223	-2.063491055	-2.540384577	-5.027296801	-1.141519639
BUB3	1.048387761	-1.478854763	-5.022493667	0.769427892
LOC729028	-1.18322571	-1.450745482	-5.022458534	-0.130175527
BMP5	-3.366166851	-2.818880256	-5.022163343	-4.393726666
LOC441873	0.807383145	0.6187226	-5.021534144	-1.353218396
SASH1	-3.591884882	-4.968887783	-5.021482067	-2.783176051
PRKCB	-2.054706642	-4.68216072	-5.01744544	-0.711448229
LOC145853	-1.12720732	-1.350068336	-5.014496266	-0.950246497
GSTM5	-3.41455243	-8.529654354	-5.013813857	-3.548005785
CD1C	-1.448023794	-1.126609706	-5.011425316	-1.066015995
ORM1	-0.207849034	-1.107377476	-5.010422133	-0.595724991
TUSC3	-1.67861206	-2.308142013	-5.008557236	-3.535852902
DHDH	-0.612774125	0.503675616	-5.006079771	-1.097068115
CTSC	-1.059024033	-1.198014133	-5.005018938	-0.33959862
LOC152578	-0.351589025	0.268256836	-5.003561663	2.770541036
SYNPO2	-0.758868181	-0.870604029	-5.002391373	-1.914880619
RFC2	-1.257166489	-1.351501404	-5.001857914	-0.621366222
