# Supplementary material for: USP15 negatively regulates lung cancer progression through the TRAF6-BECN1 signaling axis for autophagy induction
Source: Cell Death Dis. 2022 Apr 14;13(4):348. doi: 10.1038/s41419-022-04808-7 (PMC9010460; doi:10.1038/s41419-022-04808-7)
Supplement: Supplementary file 6 — Supplementary Table S3 [file 41419_2022_4808_MOESM6_ESM.pdf]

Supplementary Table 3. The list of 17 commonly up-regulated genes in four LTT tumor tissues, LTT10, LTT12, LTT26, and LTT35

TargetID (Gene)	LTT10 (Fold change, LTT10 vs. LNT10)	LTT12 (Fold change, LTT12 vs. LNT12)	LTT26 (Fold change, LTT26 vs. LNT26)	LTT35 (Fold change, LTT35 vs. LNT35)
PHF19	0.869055314	3.734664011	8.707916533	0.908472345
CCNE1	0.009361973	2.077865034	7.23083369	5.545177333
MMP9	0.849144178	3.871799099	7.117217255	6.521763813
DEPDC1B	0.25557073	1.324218451	6.97228104	1.719315259
SFN	0.727622078	3.442333083	6.164919562	1.833303577
PODXL2	0.487867911	2.48323315	4.966751986	4.047070101
KIF18A	2.155260776	3.55890279	4.474473721	0.699943241
ADAM8	1.753071198	2.573674428	4.349781449	2.6139363
UBE2C	0.483422298	1.000710382	4.345633664	3.038892154
CCR2	1.603137028	0.19364203	4.224408218	2.664219888
GALNT6	1.372630526	0.89904898	3.84639209	2.324661073
PHLDA2	5.598503102	2.732579125	3.628689527	1.828437788
FAM83A	3.199090533	3.186924007	3.580316098	8.350634551
ETV4	1.883346557	2.628710681	3.566510957	2.968531407
MYO7A	0.815599335	3.164744785	3.5088399	3.505698815
MMP11	1.953418307	2.085707617	3.165695156	5.07243987
GSDMB	2.014116008	0.755915491	3.016526482	2.712147108
