# Supplementary material for: USP15 negatively regulates lung cancer progression through the TRAF6-BECN1 signaling axis for autophagy induction
Source: Cell Death Dis. 2022 Apr 14;13(4):348. doi: 10.1038/s41419-022-04808-7 (PMC9010460; doi:10.1038/s41419-022-04808-7)
Supplement: Supplementary file 7 — Supplementary Table S4 [file 41419_2022_4808_MOESM7_ESM.pdf]

**Supplementary Table 4.** The list of 13 commonly down-regulated genes in four LTT tumor tissues, LTT10, LTT12, LTT26, and LTT35

| TargetID (Gene) | LTT10 (Fold change, LTT10 vs. LNT10) | LTT12 (Fold change, LTT12 vs. LNT12) | LTT26 (Fold change, LTT26 vs. LNT26) | LTT35 (Fold change, LTT35 vs. LNT35) |
|---|---|---|---|---|
| FMO2 | -3.14111223 | -5.830459873 | -5.328930606 | -4.490834994 |
| ZBTB16 | -3.168287093 | -3.666330223 | -4.569942575 | -4.50834101 |
| SEMA5A | -3.282331381 | -7.739205008 | -4.105211844 | -4.044076166 |
| FCN3 | -3.32114575 | -6.650156822 | -15.53550572 | -4.938997031 |
| TCF21 | -3.39640242 | -7.333840235 | -6.236978551 | -4.229944047 |
| SFTPA1B | -3.5006929 | -7.693908635 | -7.967692671 | -6.713274607 |
| HPGD | -3.546411365 | -6.745419057 | -6.736953187 | -5.031302554 |
| SOSTDC1 | -3.64226266 | -5.366039589 | -4.653026661 | -5.077314624 |
| AKAP12 | -3.818342824 | -5.533700648 | -3.906700915 | -6.528835863 |
| AFF3 | -3.915139296 | -7.928957336 | -4.609199942 | -5.32964261 |
| TMEM100 | -3.981086302 | -8.056520314 | -6.714430482 | -6.22537685 |
| GDF10 | -4.376325097 | -5.936592082 | -5.774367539 | -4.875456937 |
| WIF1 | -4.581865462 | -6.01561035 | -7.025275057 | -5.680927795 |
